# BDNF Is Associated with SFRP1 Expression in Luminal and Basal-Like Breast Cancer Cell Lines and Primary Breast Cancer Tissues: A Novel Role in Tumor Suppression?

**DOI:** 10.1371/journal.pone.0102558

**Published:** 2014-07-18

**Authors:** Laura Huth, Michael Rose, Veronika Kloubert, Wiebke Winkens, Martin Schlensog, Arndt Hartmann, Ruth Knüchel, Edgar Dahl

**Affiliations:** 1 Molecular Oncology Group, Institute of Pathology, Medical Faculty of the RWTH Aachen University, Aachen, Germany; 2 Institute of Pathology, University Hospital Erlangen, Erlangen, Germany; University of Navarra, Spain

## Abstract

Secreted frizzled related protein 1 (SFRP1) functions as an important inhibitor of the Wnt pathway and is a known tumor suppressor gene, which is epigenetically silenced in a variety of tumors e.g. in breast cancer. However, it is still unclear how SFRP1 exactly affects the Wnt pathway. Our aim was to decipher SFRP1 involvement in biochemical signaling in dependency of different breast cancer subtypes and to identify novel SFRP1-regulated genes. We generated SFRP1 over-expressing *in vitro* breast cancer models, reflecting the two major subtypes by using basal-like BT20 and luminal-like HER2-positive SKBR3 cells. DNA microarray expression profiling of these models revealed that SFRP1 expression potentially modulates Bone morphogenetic protein- and Smoothened signaling (p<0.01), in addition to the known impact on Wnt signaling. Importantly, further statistical analysis revealed that in dependency of the cancer subtype model SFRP1 may affect the canonical and non-canonical Wnt pathway (p<0.01), respectively. While SFRP1 re-expression generally mediated distinct patterns of transcriptionally induced or repressed genes in BT20 and SKBR3 cells, brain derived neurotrophic factor (*BDNF*) was identified as a SFRP1 induced gene in both cell lines. Although BDNF has been postulated as a putative oncogene, the co-regulation with SFRP1 indicates a potential suppressive function in breast cancer. Indeed, a positive correlation between SFRP1 and BDNF protein expression could be shown (p<0.001) in primary breast cancer samples. Moreover, TCGA dataset based analysis clearly underscores that *BDNF* mRNA is down-regulated in primary breast cancer samples predicting a poor prognosis of these patients. In line, we functionally provide evidence that stable BDNF re-expression in basal-like BT20 breast cancer cells blocks tumor cell proliferation. Hence, our results suggest that BDNF might rather mediate suppressive than promoting function in human breast cancer whose mode of action should be addressed in future studies.

## Introduction

The Wnt signaling pathway regulates a wide range of fundamental cellular processes in embryonic development, cell differentiation and cell proliferation [1]–[3]. Thus, it is not surprising that most human tumors exhibit features of a deregulated Wnt signaling whose enhanced activation may constitute a key feature driving the tumorigenic process of several tumor entities [4], [5]. Proteins of the “Secreted Frizzled Related Protein” family (SFRPs) are major antagonists of Wnt signaling [6]. Owing to their direct interaction with Wnt molecules via the CRD domain, SFRPs mediate the interruption of Frizzled receptor activation and therewith of intracellular mediators such as β-catenin downstream of Wnt [7], [8]. In line with that, family members of *SFRP* genes such as *SFRP1* are thought to act as tumor suppressors [9] and expression of *SFRP1* has been shown to be downregulated in many human cancer types like colorectal, breast, bladder cancer and medulloblastoma [9]–[12]. Hypermethylation of the *SFRP1* promoter that has been determined as the molecular cause of its gene silencing is a frequent event in tumorigenesis, for example in colorectal cancer [9]. In breast cancer development *SFRP1* promoter hypermethylation has been found to occur frequently (>65%) as well which is furthermore associated with unfavorable prognosis [13]. Moreover it has been clearly shown that SFRP1 re-expression led to a decreased *in vitro* tumor cell proliferation of human breast cancer cells [14]. Additionally, SFRP1 re-expressing breast cancer cells revealed a reduced tumor outgrowth *in vivo* supporting the putative tumor suppressive role of *SFRP1* [14] although detailed mechanisms of SFRP1 function and its impact on Wnt signaling in dependency of different breast cancer subtypes are still lacking.

In this study, we performed a systematic expression analysis of stably transfected human breast cancer cells to determine those molecules and biochemical pathways affected after forced SFRP1 re-expression. Subsequently, we will name these regulated molecules “SFRP1 target genes” though we are aware that most of these genes will be indirectly controlled by SFRP1 re-expression via different intracellular signaling cascades affecting nuclear transcription. The breast cancer cell lines BT20 and SKBR3 were chosen for these *in vitro* models because they do not exhibit any endogenous SFRP1 expression [13] and they belong to different molecular subgroups of human breast cancer cell lines [15]. BT20 cells are part of the basal-like gene cluster whereas HER2-positive SKBR3 cells represent the luminal cluster [15]. Still SFRP1 may confer growth-inhibitory signals in such tumor lines via independent or rather different pathways which we would like to decipher in more detail.

## Materials and Methods

### Cell culture and stable transfection

The human breast cancer cell lines BT20 and SKBR3 were obtained from the ATCC (Rockville, MD, USA) and cultured under recommended conditions. All transfections were performed using FuGene HD Transfection Reagent (Roche, Mannheim, Germany) following the manufacturer's guidelines. BT20 and SKBR3 cells were stably transfected with pEF6/V5 (Invitrogen, Carlsbad, CA, USA) encoding human SFRP1 and empty pEF6/V5. The selected stable BT20 and SKBR3 clones were maintained in complete culture medium containing 8 µg/mL and 4 µg/mL blasticidin, respectively. Moreover BT20 cells were transfected with a full-length cDNA of BDNF or empty pT-REx-DEST30 vector control (Invitrogen, Carlsbad, CA, USA) and stable clones were selected using 2.000 µg/mL G418.

### Breast cancer tissue specimens and tissue microarray (TMA)

Tumorous breast tissue samples analyzed in this study were obtained from the tumor bank of Euregional comprehensive Cancer Center Aachen (ECCA), now part of the RWTH centralized biomaterial bank (RWTH cBMB; http://www.cbmb.rwth-aachen.de). All patients gave written informed consent for retention and analysis of their tissue for research purposes according to local Institutional Review Board (IRB)-approved protocols (approval no. EK-206/09) of the medical faculty of the RWTH Aachen University. Tumor material was immediately snap-frozen in liquid nitrogen. H&E-staining of each tissue was prepared to determine the percentage of tumor cells. Only samples with more than 70% tumor cells were selected for further analysis. The tissue microarray was established at the Institute of Pathology, University of Regensburg, as described previously [16]. Data from primary breast cancer tissues and solid normal tissues were used from The Cancer Genome Atlas (TCGA) of the Ilumina mRNA expression platform (n = 1032) [17]. The data of this study can be explored using the cBio Cancer Genomics Portal (http://cbioportal.org).

### RNA isolation and real-time PCR

RNA from cell culture and primary breast tissues was extracted by use of TRIzol reagent (Invitrogen, Carlsbad, CA, USA), according to the manufacturers' recommendations. For semiquantitative real-time PCR, each sample cDNA was made from 1 µg RNA using the Reverse Transcription System (Promega, Madison, WI, USA). The IQ5 real-time PCR Detection System (Bio-Rad Laboratories, Munich, Germany) was used as described previously [18]. The primer sequences used in this study are shown in the supplements (Table S1).

### Protein extraction and Western blotting

Total cell protein extraction was performed by using lysis buffer (Invitrogen, Carlsbad, CA, USA). For Western blotting, samples were denatured for 5 min at 95°C, separated on a 4–12% polyacrylamid gel and then transferred to a nitrocellulose membrane (room temperature, 1 h, Bio-Rad). Membranes were blocked with 5% skim milk in TBS-T for 1 h at room temperature and then incubated with the first antibody overnight at 4°C. The following primary antibodies were used: anti-SFRP1 (Santa Cruz, sc-13939, CA, USA), anti-BDNF (Santa Cruz, sc-546, CA, USA), anti-LY96 (Abcam, ab24182, Cambridge, UK) and anti-β-actin (Sigma-Aldrich, A5316, Deisenheim, Germany). Afterwards membranes were washed three times with TBS-T and incubated for 1 h with horseradish peroxidase-conjugated secondary antibodies (DAKO, Glastrup, Denmark). After three washes with TBS-T, the signal was detected by chemiluminescence (Pierce ECL, Thermo Scientific, Rockford, IL, USA).

### Immunohistochemistry

Immunohistochemical analysis was performed according to the manufacturer's guidelines (EnVision Kit, DAKO K8001, Glostrup, Denmark). After deparaffinization and rehydration the tissues are heated for 30 min in 10 mM sodium citrate buffer (pH 7.2). Endogenous peroxidases are blocked by use of peroxidase blocking solution (DAKO S2023). The primary antibodies anti-SFRP1 (Pineda, Berlin, Germany) and anti-BDNF (Santa Cruz sc-546, CA, USA) were applied for 1 h at room temperature and overnight at 4°C, respectively. Intensity of the immunohistochemical staining was scored by an experienced pathologist.

### Proliferation XTT assay

For proliferation analysis the XTT cell proliferation kit II from Roche (Mannheim, Germany) was used. 1,000 cells per well were plated and 100 µl of complete culture medium were added. Proliferation was determined at four different time points: 24, 48, 72 and 96 h after incubation. 50 µl of XTT reagent solution were added to each well and afterwards incubated for 4 h. Finally the absorbance was measured at 492 nm.

### Microarray analysis

Gene expression analysis of the SFRP1 test set (SKBR3/SFRP1 and BT20/SFRP1 cells) and the control set (SKRB3/mock and BT20/mock cells) was carried out by the IZKF Chip-Facility (Interdisciplinary Centre for Clinical Research Aachen within the medical faculty of the RWTH Aachen University). Biotinylated, fragmented cRNA of stably transfected breast cancer cells was hybridized to Affymetrix U133 plus 2.0 human GeneChips (Affymetrix, Santa Clara, CA, USA). In excess of 54,000 probe sets are used to analyze the expression level of more than 47,000 transcripts and variants. The microarray data from this publication have been submitted to the European Bioinformatics Institute (EMBL-EBI) database (http://www.ebi.ac.uk/) and are available under accession number E-MTAB-2209.

### Statistical data analysis

Statistical analysis was carried out using SPSS 19.0 (SPSS, Chicago, IL, USA) and GraphPad Prism 5.0 (GraphPad Software Inc., La Jolla, CA, USA). Differences with a p-value <0.05 were defined to be statistically significant. A two-tailed Mann-Whitney U test was used to determine differences in the expression levels and the proliferation rate. Furthermore, a correlation between SFRP1 and BDNF expression was tested using Spearman correlation analysis and a two-sided Fisher's exact test.

Gene expression analyses were performed using BRB-ArrayTools developed by Dr. Richard Simon and BRB-ArrayTools Development Team version 4.3.0 – Beta. In order to significantly identify genes differentially expressed among two classes the *class comparison* evaluation was used [19]. Exact permutation p-values for significant genes were computed based on 35 available permutations. Difference of gene expression was considered significant when both conditions were achieved: i) p-values were equal or below 5% and ii) a minimum of 2-fold difference in expression level was observed. Genes were excluded when less than 20% of expression data have at least a 1.5-fold change in either direction from gene's median value. An annotation of the gene subset in Gene Ontology was geared to following conditions: GO classes and parent classes with at least 5 observations in the selected subset and with an ‘Observed *vs.* Expected’ ratio of at least 2. In order to evaluate and annotate gene lists of differential expression of Gene Ontology (GO) categories relating the whole array data, a gene set comparison analysis was performed that is similar to the gene set enrichment analysis described by Subramanian et al. [20]. Tests used to find significant gene sets were: LS/KS permutation test (to find gene sets which have more genes differentially expressed among the phenotype classes than expected by chance) and Efron-Tibshirani's GSA maxmean test (to identify gene sets differentially expressed). Over-represented GO lists were considered significant when the threshold of determining significant gene sets is equal or below 0.005 (LS/KS permutation test) or 0.05 (Efron-Tibshirani's GSA maxmean test).

Based on TCGA data set of the Ilumina mRNA expression platform [17] influence of BDNF expression on RFS was measured from surgery until local or distant relapse and was censored for patients alive without evidence of relapse at the last follow-up. Multivariate Cox-regression analysis was carried out to test for an independent prognostic value of BDNF expression. Using the KMPLOT data set Kaplan-Meier analyses of the SFRP1 co-expressed gene *BDNF* was performed as described previously [21], [22].

## Results

### Identification of gene pattern associated with luminal and basal-like breast cancer cells

**Figure 1:**
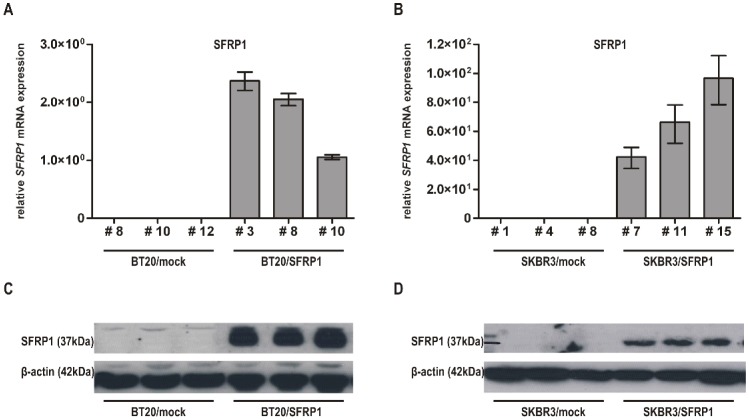
Generation of human breast cancer cell lines stably expressing SFRP1. Stable clones have been generated with a full-length SFRP1 cDNA or with empty pEF6/V5 vector control. (A) Semi-quantitative real-time PCR for SFRP1 re-expression was performed after transfection in BT20 and (B) SKBR3 cells. *SFRP1* mRNA was only detectable in the SFRP1 clones. (C) Western blot analysis was performed on lysates of three BT20 and (D) SKBR3 mock and of three SFRP1 clones. SFRP1 protein expression increased remarkably after transfection with a SFRP1 expression vector compared to the corresponding mock vector. β-actin was used as a loading control.

First, two human breast cancer cell lines lacking *SFRP1* mRNA [13] were selected to generate breast cancer *in vitro* models exhibiting forced SFRP1 re-expression: the basal-A breast cancer cell line BT20 and the luminal-like HER2-positive breast cancer cell line SKBR3 [15]. Expression analysis of BT20 and SKBR3 mock and SFRP1 clones confirmed that *SFRP1* mRNA levels increased remarkably in the SFRP1 clones (Figure 1A and B). In stable transfected BT20/SFRP1 and SKBR3/SFRP1 cells, SFRP1 was strongly re-expressed compared to the mock controls which completely lack SFRP1 protein (Figure 1C and 1D). Thus, a successful generation of SFRP1 over-expressing BT20 and SKBR3 breast cancer cells could be shown on mRNA and protein level. Figure 1 exemplarily compares three mock and three SFRP1 clones of each manipulated breast cancer cell line.

### Defining novel SFRP1 target genes

**Figure 2:**
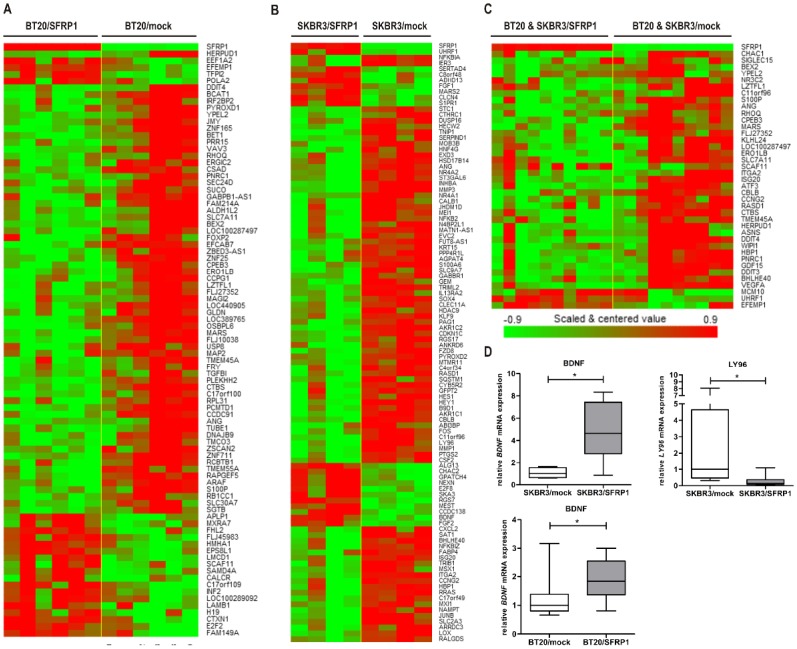
Microarray analysis from stable transfected cell lines BT20 and SKBR3. Genes represented have a p value of 0.05 or less and are regulated at least ±2-fold. (A) 87 differentially expressed genes were found by comparing BT20/SFRP1 and BT20/mock cells. (B) Comparison of SKBR3/SFRP1 and SKBR3/mock cells revealed 104 differentially expressed genes. (C) By applying class comparison between SFRP1 clones (BT20 and SKBR3) and mock clones (BT20 and SKBR3) 40 differentially expressed genes were discovered. (D) Validation of SFRP1 target genes in SKBR3 and BT20 model system. Semi-quantitative real-time PCR was performed for each target gene in the particular *in vitro* model. *BDNF* mRNA levels increased in SKBR3/SFRP1 clones compared to the mock controls (p<0.05). In contrast *LY96* mRNA was up-regulated in SKBR3 mock clones (p<0.05). BT20 cells showed an increase of *BDNF* mRNA levels after SFRP1 re-expression (p<0.05).

To identify genes affected by SFRP1 re-expression either in association with a distinct breast cancer subtype, or independently of those, we performed a comprehensive whole human genome expression and compared identified pattern. Based on the luminal SKBR3 and the basal-A BT20 gain-of-function *in vitro* model, the expression level of 47.000 transcripts and variants was evaluated using the Human Genome U133 plus 2.0 array. In case of basal-A BT20, our analysis revealed 87 genes that were differentially expressed by a factor of 2.0 or more and showed a significant p value (p<0.05) between mock and SFRP1 transfected BT20 cells (Table S2). Of these 87 genes, 23 were up-regulated and 64 were down-regulated due to SFRP1 re-expression (Figure 2A). Following identical criteria we found overall 104 differentially expressed genes in the SKBR3/SFRP1 clones compared to SKBR3 mock clones. The lists of these SFRP1 target genes are given in the Supporting Information (Table S2 and S3). Results of the SKBR3 microarray data revealed 20 up-regulated and 84 down-regulated genes in the SFRP1 re-expressing SKBR3 clones (Figure 2B). To further reveal putative target genes of SFRP1 which are affected independently of a given subtype, we statistically evaluate gene patterns based on both *in vitro* models. By applying a class comparison analysis between control cell populations combining SKBR3/ and BT20/mock clones) and SFRP1 expressing SKBR3/ and BT20/SFRP1 clones, only a small number of 40 genes was detected to be significantly regulated by SFRP1 expression calculated dependent on the same stringent criteria. (Figure 2C).

The microarray expression profiling revealed various SFRP1 associated genes (see Figure 2). In order to define now novel target genes of SFRP1, the first criteria for gene selection was the confirmation of a differently expression of these identified target genes. By real-time PCR we validated candidate gene *LY96* transcript that was significantly down-regulated in SFRP1 expressing SKRB3 cells when compared to controls (Figure 2D). Interestingly, the Brain-derived neurotropic factor (*BDNF*) gene transcript was shown to be co-expressed with SFRP1 in both subtypes, i.e. in the luminal SKBR3 as well as the basal-A BT20 cells (Figure 2D).

### Annotation of biological processes affected by SFRP1 expression in luminal and basal-A breast cancer cell lines

In order to better understand SFRP1 function and biological processes involved in SFRP1 mediated target gene modulation in dependency of a distinct breast cancer subtype we performed a gene ontology analysis (GO) in the BT20 and SKBR3 tumor models (Table 1 and 2). An influence of SFRP1 expression on the regulation of the BMP- and Smoothened signaling pathways could be concordantly demonstrated in both tumor models, luminal-like SKBR3 and basal-like BT20. Moreover a connection between angiogenesis and SFRP1 expression was also observed. Interestingly, the main difference in the GO analysis between both tumor models was the association to the Wnt signaling pathway. In the luminal-like SKBR3 tumor model the SFRP1 expression had an influence on the regulation of the non-canonical Wnt signaling pathway (p<0.01). By contrast in the basal-like BT20 cells the SFRP1 expression showed an association with the canonical Wnt signaling pathway (p<0.01). Interestingly, GO analysis between control cell populations (SKBR3/ and BT20/mock clones) and SFRP1 clones (SKBR3/ and BT20/SFRP1 clones) revealed a general influence of SFRP1 expression on the regulation of the Wnt signaling pathway (Table 3; p<0.05) whereas no distinct association with the canonical or the non-canonical-pathway was statistically detectable. Instead, an involvement of SFRP1 in BMP- and Smoothened signaling could be suggested for both cell lines: we found a highly statistical likelihood of this association with respect to both tests, i.e. considering the “LS permutation p-value” (p<0.01) and the “Efron-Tibshirani's GSA test p-value” (p<0.05). These findings indicate a biochemical pathway mechanism that is potentially independent of the mirrored breast cancer subtype.

**Table 1:** Selected GO categories of the microarray analysis of the basal-like BT20 tumor model.

GO ontology	GO category	GO term	Number of genes	LS permutation p-value	KS permutation p-value	Efron-Tibshirani's GSA test p-value
BP	GO:0045785	positive regulation of cell adhesion	6	**0.00262**	0.20461	**<0.005 (−)**
BP	GO:0045892	negative regulation of transcription, DNA-dependent	29	**0.0019**	0.12585	0.095 (−)
BP	GO:0043065	positive regulation of apoptotic process	30	**0.00543**	0.05681	**0.03 (+)**
BP	GO:0045765	regulation of angiogenesis	8	**0.00704**	0.19637	**<0.005 (−)**
BP	GO:0030308	negative regulation of cell growth	9	**0.00732**	0.23562	0.06 (−)
BP	GO:0090090	negative regulation of canonical Wnt receptor signaling pathway	7	**0.0076**	0.57791	**0.005 (−)**
BP	GO:0060828	regulation of canonical Wnt receptor signaling pathway	9	**0.01344**	0.59123	**0.025 (−)**
BP	GO:0030509	BMP signaling pathway	8	**0.0143**	0.8705	**0.005 (−)**
BP	GO:0021915	neural tube development	9	**0.01324**	0.6942	**<0.005 (−)**
BP	GO:0001525	angiogenesis	18	**0.0239**	0.44111	0.06 (−)
BP	GO:0030111	regulation of Wnt receptor signaling pathway	14	**0.02798**	0.63057	0.085 (−)
BP	GO:0007224	smoothened signaling pathway	5	**0.00353**	0.31383	**<0.005 (−)**

**Table 2:** Selected GO categories of the microarray analysis of the luminal-like SKBR3 tumor model.

GO ontology	GO category	GO term	Number of genes	LS permutation p-value	KS permutation p-value	Efron-Tibshirani's GSA test p-value
BP	GO:0001525	angiogenesis	91	**0.00001**	**0.00052**	**0.005 (+)**
BP	GO:0030510	regulation of BMP signaling pathway	10	**0.0004**	**0.03264**	**<0.005 (−)**
BP	GO:0007224	smoothened signaling pathway	11	**0.00084**	0.17282	**0.005 (−)**
BP	GO:2000050	regulation of non-canonical Wnt receptor signaling pathway	5	**0.00033**	0.2385	**<0.005 (−)**
BP	GO:0060071	Wnt receptor signaling pathway, planar cell polarity pathway	7	**0.00058**	0.36931	**<0.005 (−)**
BP	GO:0001841	neural tube formation	32	**0.00136**	0.16301	**<0.005 (−)**
BP	GO:0032870	cellular response to hormone stimulus	81	**0.00007**	0.07944	0.125 (+)

**Table 3:** Selected GO categories of the microarray analysis independent of the different breast cancer subtypes.

GO ontology	GO category	GO term	Number of genes	LS permutation p-value	KS permutation p-value	Efron-Tibshirani's GSA test p-value
BP	GO:0071772	response to BMP stimulus	5	**0.00034**	0.25838	**<0.005 (−)**
BP	GO:0030178	negative regulation of Wnt receptor signaling pathway	58	**0.0321**	0.49944	**0.025 (−)**
BP	GO:0030510	regulation of BMP signaling pathway	30	**0.01627**	0.60267	**0.005 (−)**
BP	GO:0007224	smoothened signaling pathway	28	**0.00172**	0.21086	**0.005 (−)**
BP	GO:0014020	primary neural tube formation	40	**0.00914**	0.3122	**<0.005 (−)**

### Correlation analysis of SFRP1 and BDNF protein expression in primary breast cancer

**Figure 3:**
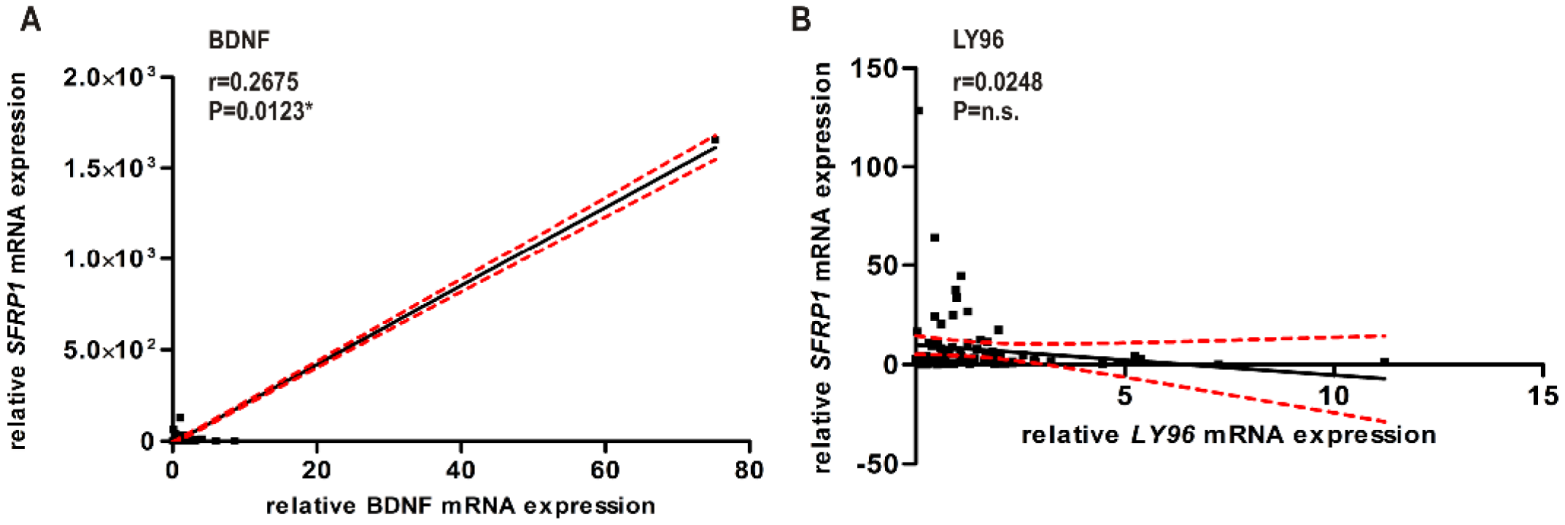
Correlation analysis of mRNA expression in human breast cancer tissues. (A) By comparing the *BDNF* and *SFRP1* mRNA expression of 87 tumor samples a significant correlation was found (Spearman coefficient, p<0.05). (B) A correlation between LY96 and SFRP1 mRNA expression in 86 human breast cancer tissues could not be detected. n.s.: not significant.

Next, we wanted to decipher whether the positive correlation between SFRP1 and BDNF expression on the one hand and the negative correlation between SFRP1 and LY96 expression on the other hand is also detectable in human breast cancer. To that end we analyzed SFRP1 expression in 85 breast cancer tissue samples by real-time PCR. Afterwards the *BDNF* and *LY96* mRNA levels were determined in the same tumor tissue samples and compared to the SFRP1 expression level in the particular tumor sample. Most interestingly, this correlation analysis (Spearman) of mRNA expression levels revealed that *SFRP1* and *BDNF* mRNA expression showed a significant correlation *in vivo* (p<0.05; Figure 3A). However, an *in vivo* correlation of SFRP1 and LY96 could not be detected (Figure 3B). Thus, LY96 was excluded from further analysis. In summary we assessed that forced SFRP1 expression in BT20 and SKBR3 breast cancer cells leads to an induction of BDNF expression. This positive correlation *in vitro* may be maintained *in vivo* due to the tight correlation detected between *SFRP1* and *BDNF* mRNA in human breast cancer samples.

**Figure 4:**
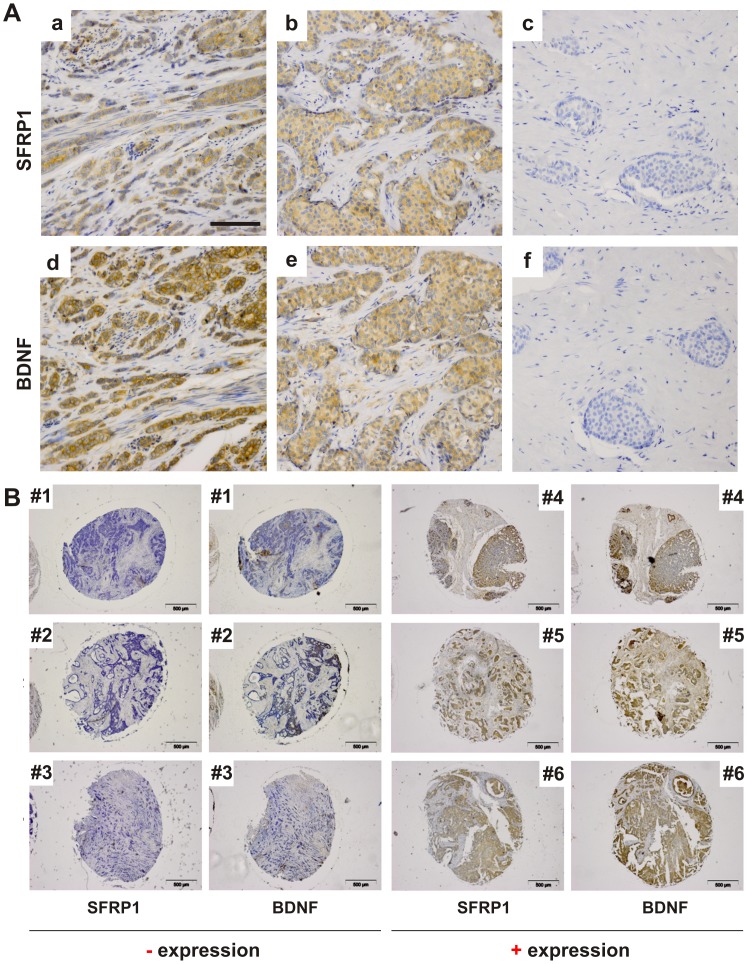
Analysis of SFRP1 and BDNF expression in human breast tissues. (A) Immunohistochemical staining of human breast tissues is shown exemplarily. SFRP1 and BDNF protein expression of tissue samples from the same patient (a and d, b and e) is shown. c and f: negative controls of SFRP1 and BDNF immunohistochemical staining, respectively. Scale bar: 100 µm. (B) In the left columns three tissues (patient #1 - #3) exhibit no or weak staining of SFRP1 and BDNF whereas the right columns represent three tissues (patient #4 - #6) that have a strong SFRP1 and BDNF protein expression. These images illustrate exemplarily the correlation of SFRP1 and BDNF protein expression *in vivo*. Scale bars: 100 µm.

It was a clear objective to subsequently study whether the correlation between SFRP1 and BDNF expression in primary breast cancer samples is preserved on the protein level as well. Therefore, we performed immunohistochemical staining of SFRP1 and BDNF on a breast cancer tissue microarray comprising 144 specimens. Indeed, again we found a very strong correlation (p<0.0001; Table 4) between SFRP1 and BDNF protein expression. Tumor samples lacking SFRP1 protein expression also exhibited loss of BDNF protein expression and vice versa. This can be exemplary shown in six subsequent tissue samples stained with antibodies directed against SFRP1 and BDNF proteins, respectively (Figure 4A and 4B). Thus, the immunohistochemical analysis of human breast cancer specimens again confirmed that BDNF is a potential “SFRP1 target gene” as originally predicted by our microarray analysis. This could indeed indicate that there is a physiological relevance for SFRP1-BDNF expression axis found in human breast cancers.

**Table 4:** Correlation analysis of immunohistochemical staining of SFRP1 and BDNF protein.

		SFRP1 protein expression		Pearson correlation	Significance (two-sided)
BDNF protein expression	n	low (0–1)	high (2–3)		
low (0–1)	46	31	15	0.401	0.000*
high (2–3)	98	25	73		

### BDNF protein loss in human breast cancer and its clinical impact on recurrence-free survival

**Figure 5:**
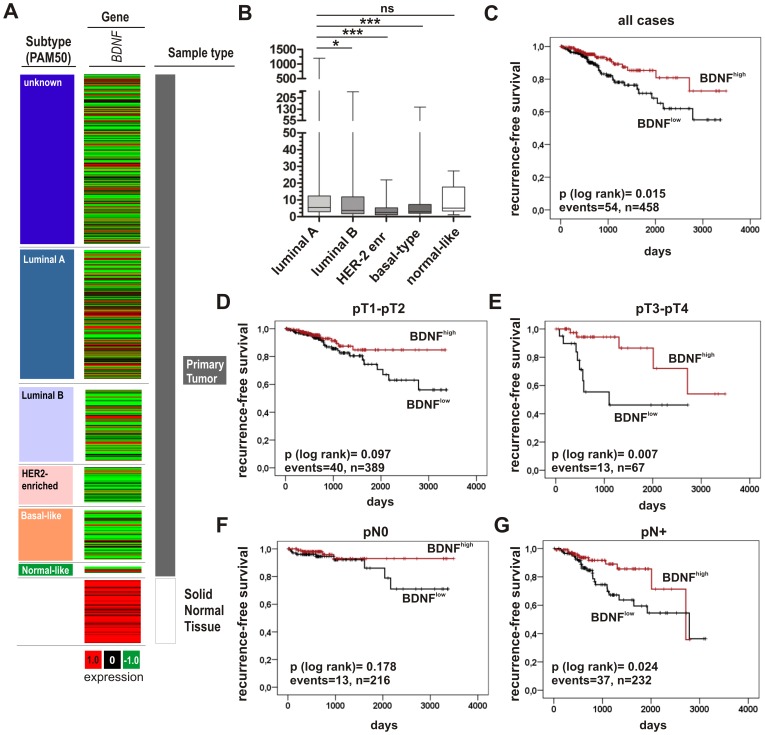
*BDNF* gene expression is associated with longer recurrence-free survival in human breast cancer of public TCGA data sets [17]. (**A**) *BDNF* expression in breast tumor samples. Red: high expression, black: mean expression and green: low expression. *Left panel*: clinical data. *Middle panel*: *BDNF* mRNA expression. *Right panel*: sample type (dark grey: primary tumor; white: solid normal tissues; based on TCGA Ilumina platform, n = 1032 samples). (**B**) *BDNF* expression in relation to tumors stratified by subtypes [23]. *ns*: not significant, *p<0.05, ***p<0.001. (**C–G**) Kaplan-Meier analysis of the TCGA data set illustrating RFS of patients with high *BDNF* (red curve) compared to reduced *BDNF* expression (black curve) in (**C**) all, (**D**) pT1-pT2 (**E**) pT3-pT4 (**F**) nodal-negative (pN0) or (G) nodal-postive (pN+) breast cancer patients. Vertical lines: censored cases.

Given that BDNF is induced by forced SFRP1 re-expression in both luminal-like SKBR3 and basal-like BT20 tumor cells, we hypothesized a putative tumor suppressive role of BDNF. To assess the potential relevance of this hypothesis, we analyzed *BDNF* gene expression and its clinical impact in a large dataset of independent studies [17], in total representing 3.910 different breast cancer samples. Using data of The Cancer Genome Atlas (TCGA), a prevalent loss of *BDNF* gene expression in breast tumors when compared to normal breast tissues could be confirmed (Figure 5A). Stratifying this TCGA data set [17] by defined breast cancer subtypes based on Hu et al. [23] we demonstrated a pronounced loss of *BDNF* mRNA in subtypes associated with poor prognosis, i.e. luminal B, HER2-enriched and basal-like breast cancer (Figure 5B). In this public data set, univariate analyses showed that reduced *BDNF* expression is significantly associated with a shorter RFS (Figure 5C), in particular in clinical important groups of patients with advcanced tumor stages (Figure 5D–G). The calculated Cox regression model, including all factors potentially relevant to influence RFS available in this data set, highlighted a strong impact of BDNF expression on early recurrence (Table 5). Breast cancer patients with low BDNF expression have an decreased risk for tumor relapse compared with patients with high BDNF expression (multivariate hazard ratio (HR): 0.374, p<0.05). Hence, BDNF loss notably increased the risk for early tumor relapse.

**Table 5:** Multivariate Cox regression analysis including all factors potentially influencing RFS.

Variable	HR	P-value	95%CI	
			lower	upper
*BDNF* mRNA *expression* a				
BDNF^low^	1.000			
BDNF^high^	0.374	**0.015**	0.170	0.824
Tumour size^b^				
pT1-pT2	1.000			
pT3-pT4	1.863	0.133	0.827	4.195
Lymph node status^b^				
pN0	1.000			
pN1-3	2.174	0.054	0.988	4.783
Oestrogen receptor status^b^				
negative	1.000			
positive	1.941	0.184	0.730	5.164
Progesterone receptor status^b^				
negative	1.000			
positive	0.340	**0.017**	0.141	0.823
HER2 status^b^				
negative	1.000			
positive	0.443	0.193	0.130	1.509

a Median *BDNF* mRNA expression = 4.25 (low: ≤ median expression, high: >median expression);

b According to clinical data of the TCGA data set [17]; Significant P-values are marked in bold face.

**Figure 6:**
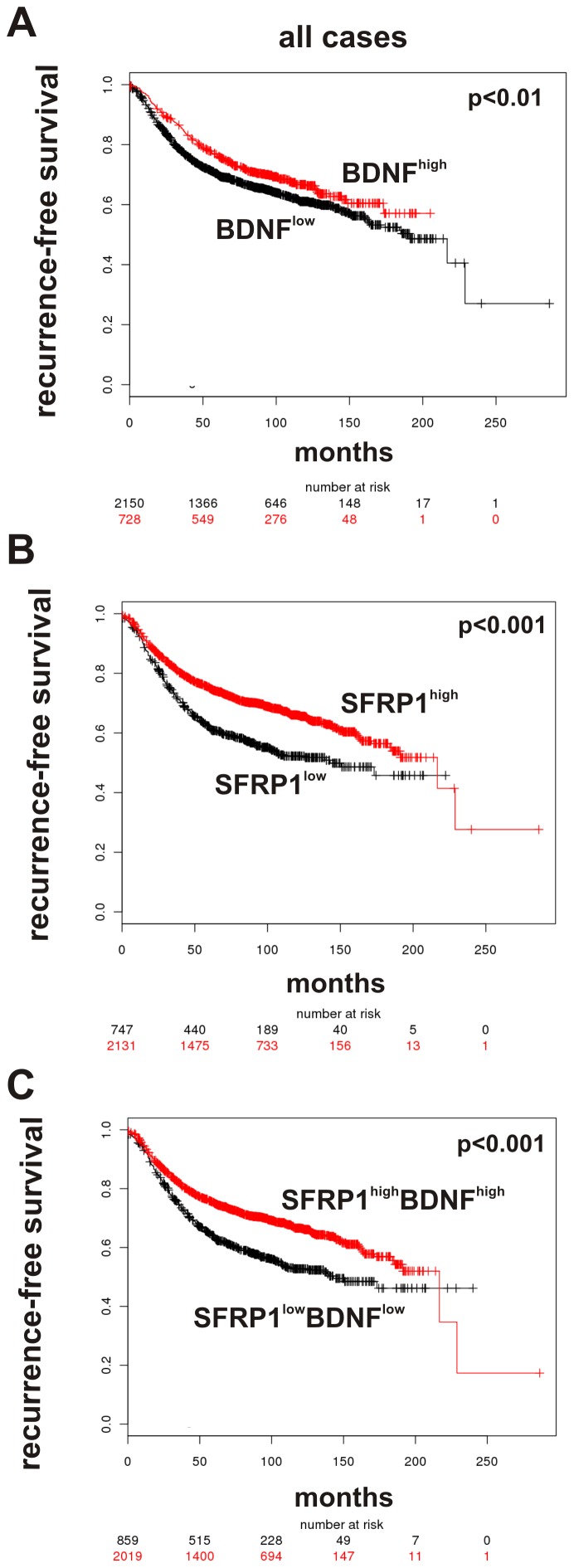
The clinical impact of *SFRP1/BDNF* gene expression in human breast cancer. Using the KMPLOT data set an association between unfavorable clinical outcome for breast cancer patients and (**A**) *SFRP1* expression, as well as (**B**) *BDNF* expression and (**C**) a combination of abundant *SFRP1/BDNF* expression was observed.

Next, we confirmed in a further independent data set of 2.878 breast tumors (KMPLOT) the known prognostic impact of BDNF expression on recurrence-free survival (RFS) (Figure 6A). As published so far, reduced *SFRP1* expression was significantly associated with unfavorable clinical outcome for breast cancer patients as well (Figure 6B). Importantly, a combination of abundant expression of *SFRP1* and *BDNF* was clearly correlated with longer RFS (p = 0.0019) in the KMPLOT data set as well (Figure 6C), underscoring a putative clinical impact of the SFRP1-BDNF expression axis.

### BDNF over-expression in basal-A BT20 breast cancer cells suppresses tumor growth *in vitro*

**Figure 7:**
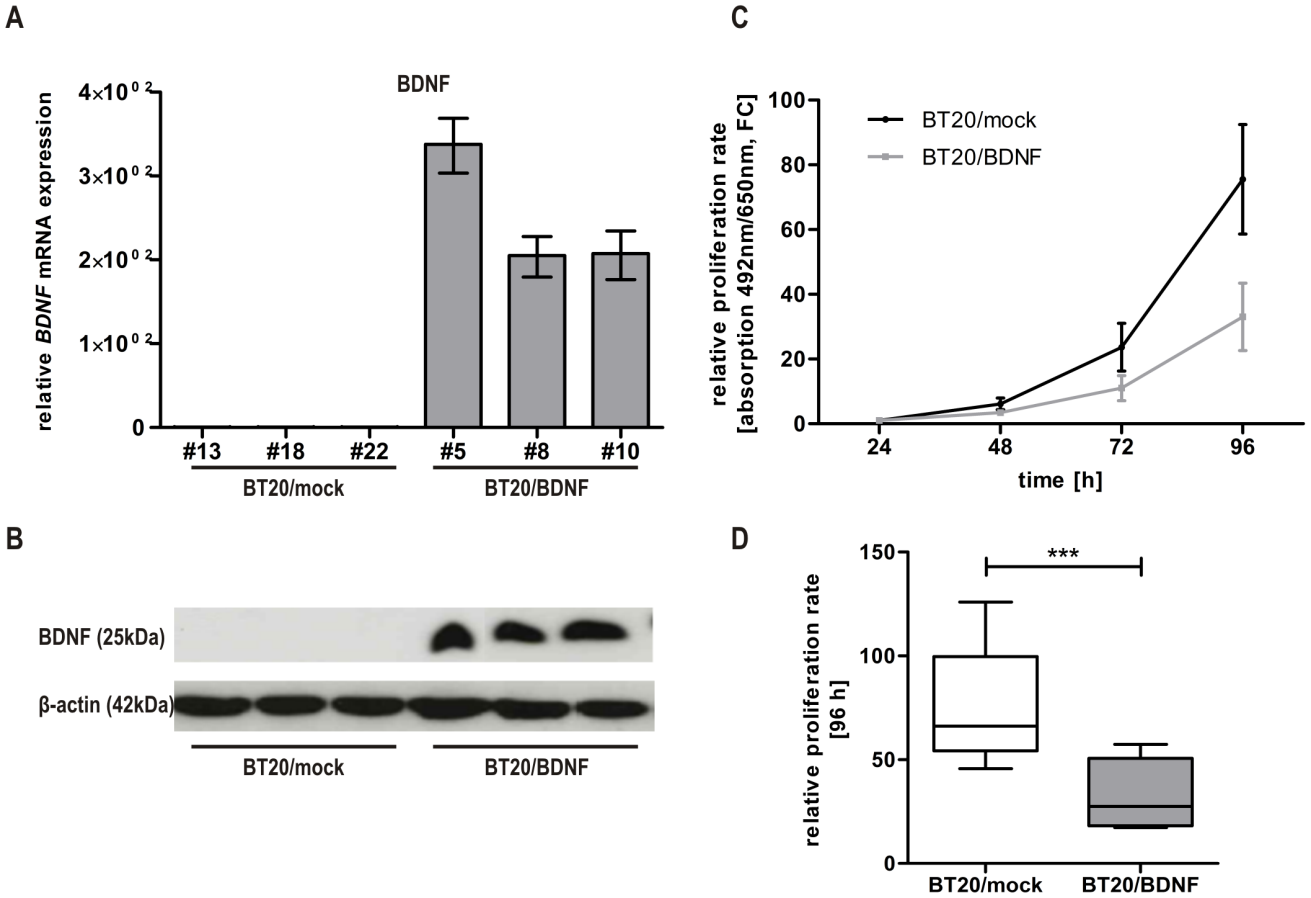
BDNF re-expression mediates reduced cell proliferation in BT20 breast cancer cells. (A) Stable cell clones with a full-length cDNA of *BDNF* show abundant re-expression of *BDNF* mRNA while empty pT-Rex-DEST30 vector controls completely lack *BDNF* mRNA. (B) In concordance, mock clones are negative for BDNF protein whereas the 25 kDa BDNF protein is strongly expressed in stable BDNF clones. (C) XTT assay was performed at four subsequent time points. The baseline level at 24 h for each clone was set to 1. A slight decrease in cell proliferation (58.4%) was observed in the stable BDNF clones. (D) Proliferation is significantly (p<0.001) reduced in BT20 breast cancer cells re-expressing BDNF.

In contrast to recent published studies highlighting *BDNF* as a putative oncogene [24], the Kaplan-Meier data of the independent patient data set suggested rather suppressive characteristics of BDNF in breast cancer development. To determine, whether BDNF, a potential novel target gene of the tumor suppressor SFRP1, may also mediate inhibiting effects on cell proliferation of breast cancer cells, we generated a stable BDNF over-expressing breast cancer *in vitro* model using BT20 breast cancer cells. Expression analysis of BT20/mock and BT20/BDNF clones confirmed that *BDNF* mRNA levels increased significantly in the BT20/BDNF clones (Figure 7A). Concordantly, expression of BDNF protein in the BDNF-transfected BT20 clones and lack of BDNF protein expression in the mock-transfected clones could be verified by western blot analysis (Figure 7B). Subsequently, a proliferation assay was performed for three independent BDNF re-expressing BT20 clones and three independent mock clones using the XTT assay. The optical density measured at the first time point (24 h) was used for normalization. Indeed, we observed a decreased mean proliferation level after BDNF re-expression at the time points 72 and 96 h (Figure 7C). Thus, cellular proliferation was significantly (p<0.001) reduced in basal-like BT20 clones which stably expressing BDNF protein (Figure 7D).

## Discussion

A key feature of human breast cancer is the presence of aberrant Wnt signaling activation [1]. The putative tumor suppressor SFRP1 is an important inhibitor of this signaling pathway [3], [4]. Consequently a high frequency of human breast tumors shows hypermethylation and transcriptionally silencing of the *SFRP1* gene [13]. Nevertheless, too little is known about signaling pathways modulated by SFRP1 expression in dependency of the molecular breast cancer subtypes. To address this question we initiated a systematic whole genome expression analysis to determine biochemical pathways as well as novel target genes that are affected by SFRP1 in both luminal-like and basal-like breast cancer cells.

Based on SKBR3 and BT20 *in vitro* models we initially characterized gene signatures and signaling pathways modulated by forced SFRP1 re-expression. SFRP1 caused subtype specific signatures. For example down-regulation of the Lymphocyte antigen 96 (LY96), a 25 kDa co-receptor of the LPS signaling, that plays a critical role in immune response, [25] was demonstrated solely in luminal SKBR3 cells. Overall, we detected 87 and 104 genes which were differentially expressed in mock and SFRP1 transfected BT20 and SKBR3 cells, respectively, while 40 genes were affected independently of the subtype analyzed. Of interest distinct Wnt signaling pathways may be triggered by SFRP1 in dependency of different breast cancer subtypes. Our *in silico* data clearly showed that in basal-like BT20 tumor cells SFRP1 is involved in modulating the canonical Wnt pathway. Contrary to that in luminal SKBR3 cells the non-canonical Wnt pathway was significantly influenced by SFRP1. Most interestingly, SFRP1 seems to be involved in BMP signaling cascade in both tumor models, i.e. independently of the mirrored subtype. The bone morphogenetic proteins (BMPs) are paracrine signaling molecules of the TGF-β signaling pathway [26]. Binding of BMPs to membrane receptors results in an intracellular signaling cascade and leads ultimately to phosphorylation of Smad proteins, which act as transcription factors in the nucleus [27]. Interestingly, the interaction of TCF with Smad 4 is thought to connect BMP with the Wnt signaling pathways [28]. In line with these data, loss of SFRP1 has been associated with a direct role in the TGF-β signaling pathway [29]: Down-regulation of SFRP1 in a non-malignant breast cell line resulted in an increase of TGF-β-target molecules. Further data of Labbé and colleagues suggest an association between the Wnt and TGF-β signaling promoting tumor development [30]. The detailed mechanistic linkage is unknown but it has been demonstrated that in cells with an active Wnt signaling β-catenin was translocated into the nucleus. As a result it has been further shown that β-catenin interacts with the transcription factor LEF1, which is in turn associated with Smad 4, thereby regulating the transcription of TGF-β target molecules [31]. In concordance with these data, our findings propose an inhibiting impact of the Wnt inhibitor SFRP1 on the BMP/TGF-β associated pathway.

Among the identified genes transcriptionally affected by SFRP1, we clearly characterized brain-derived neurotrophic factor (*BDNF*) as a putative co-expressed gene. BDNF belongs to the neurotrophin superfamily of polypeptide growth factors and plays important roles in neuronal survival, neurogenesis, differentiation and neurite growth throughout the central nervous system [32]. Moreover BDNF has been associated with various human diseases including depression, epilepsy, Alzheimer, Parkinson and Huntington [33], [34], i.e. BDNF has been mainly studied as a growth factor in the central and peripheral nervous system. However, its possible role in tumorigenesis came to the focus of research in recent years. By now, BDNF has been studied in several human cancers: neuroblastoma, ovarian, lung, prostate cancer (rather as an oncogene) [35]–[39] and breast cancer [32]. In the latter Vanhecke and colleagues recently investigated BDNF expression pattern. They showed that BDNF is expressed and secreted in breast cancer cells and functionally contributes to cancer cell survival. An anti-BDNF treatment in xenografted mice resulted in tumor growth inhibition [32]. In parallel researchers revealed that BDNF expression is significantly increased in breast cancer tissues compared to normal tissues and may be associated with unfavorable pathological parameters [23], [40]. By contrast Blasco-Gutiérrez et al. were not able to detect any differences in the BDNF expression between tumor and normal tissues [41], hence the significance of BDNF expression in breast cancer remains still unclear.

Our *in vitro* results indicate that BDNF is up-regulated due to SFRP1 expression in BT20 as well as in SKBR3 cells. Bearing in mind that *SFRP1* is a known tumor suppressor gene [13], [14] this association gives insight into a novel putative BDNF function. A direct coherence of the identified BDNF/SFRP1 expression axis is further supported by the significant correlation of BDNF and SFRP1 protein expression in primary breast tumors. In agreement with that independent public data set analysis revealed an abundant loss of *BDNF* expression in primary breast cancer tissues compared with normal breast tissues. Interestingly, *BDNF* loss was abundantly found in breast cancer subtypes associated with poor prognosis, i.e. luminal B, HER2-enriched and basal-like breast cancer. In agreement with that, BDNF expression loss was further associated with poor prognosis, i.e. shorter recurrence-free survival. These findings underscore the importance of *BNDF* inactivation in breast cancer and rather supporting putative suppressive capabilities of BDNF seen in our *in vitro* experiments. These data are contradictory to Patani's work [24] wherefore this inconsistency has to be clarified in future studies. However, combined analysis of weak *BDNF* and weak *SFRP1* expression predicted also an unfavorable patients' outcome underscoring the significance of the breast cancer model-based SFRP1/BDNF expression axis.

Further analysis of the functional impact of BDNF on basal-like BT20 breast cancer cells supported our hypothesis that BDNF might rather mediate growth inhibiting than promoting properties. In fact, stable BDNF over-expression in BT20 basal-like cancer cells caused a reduction of tumor cell proliferation. Therewith, we propose at this step a putative suppressive function of BDNF for this clinical important subgroup of basal-like breast cancer. Thus, our data revealed novel clinical and functional characteristics of BDNF. However, Yang and co-workers recently demonstrated that a knockdown of BDNF resulted in a reduced cell proliferation in the breast cancer cell line MDA-MB231 [42]. The converse functional data might be caused by the fact that BDNF expression is down-regulated in luminal as well as basal A breast cancer cells while mesenchymal basal B breast cancer cells such as MDA-MB231 showed increased expression levels (GOBO data set analysis, data not shown). These illustrated discrepancies underline a complex function potentially due to different breast cancer subtypes that have to be addressed in future studies. However, our *in vitro* and *ex vivo* data are in concordance with the BDNF expression found in public data sets underscoring a putative suppressive function of BDNF in human breast cancer and a potential association to the Wnt pathways. According to that Yi and colleagues demonstrated that Wnt signaling induces BDNF expression in neurons and glia without understanding the underlying mechanisms [43]. Furthermore seven binding sites for the Wnt-dependent transcription factors TCF/LEF has been identified within the BDNF promoter region [43] and in a recent study BDNF has been postulated to regulate the expression of target molecules within the canonical Wnt signaling pathway [44]. Hence, these data generated in our work in comparison with recent publications illustrate a large discrepancy with respect to the function of BDNF in breast cancer. Further studies might be of interest to in depth analyze BDNF function in human breast cancer subtypes helping to explain the striking contradictions.

In conclusion, SFRP1 re-expression caused distinct gene signature pattern in dependency of a given breast cancer subtype, potentially by affecting either the canonical Wnt pathway in basal-like breast cancer cells or the non-canonical Wnt pathway in luminal-like breast cancer cells. Furthermore, to our best knowledge the current findings propose for the first time that BDNF is a target gene of SFRP1 with putative suppressive characteristics in breast cancer, thus highlighting its clinical relevance in a completely different and novel way.

## Supporting Information

Table S1 **Primer sequences used in this study.**
(DOC)

Table S2 **Detailed gene list of the 87 gene signature of basal-A BT20 cells.**
(DOC)

Table S3 **Detailed gene list of the 104 gene signature of luminal SKBR3 cells.**
(DOC)
