# Supplementary material for: BDNF Is Associated with SFRP1 Expression in Luminal and Basal-Like Breast Cancer Cell Lines and Primary Breast Cancer Tissues: A Novel Role in Tumor Suppression?
Source: PLoS One. 2014 Jul 18;9(7):e102558. doi: 10.1371/journal.pone.0102558 (PMC4103839; doi:10.1371/journal.pone.0102558)
Supplement: Table S1 — Primer sequences used in this study. (DOC) [file pone.0102558.s001.doc]

**Supporting Information S1**

**Table S1**. Primer sequences used in this study.

| **Primer** | **Sequence (5´ 🡪 3´)** | **T_A_** | **Cycle** | **Product** |
| --- | --- | --- | --- | --- |
| GAPDH_F | GAA GGT GAA GGT CGG AGT CA | 60°C | 40 | 108 bp |
| GAPDH_R | AAT GAA GGG GTC ATT GAT GG |
| SFRP1_F | AGA TGC TTA AGT GTG ACA AGT TCC | 60°C | 40 | 130 bp |
| SFRP1_R | TCA GAT TTC AAC TCG TTG TCA CAG |
| BDNF_F | AAA CAT CCG AGG ACA AGG TG | 60°C | 40 | 249 bp |
| BDNF_R | AGA AGA GGA GGC TCC AAA GG |
| LY96_F | TTC CAC CCT GTT TTC TTC CA | 57,1°C | 40 | 167 bp |
| LY96_R | TGT GCA ATA ATC CTT TGG ATC TT |
