# Supplementary material for: BDNF Is Associated with SFRP1 Expression in Luminal and Basal-Like Breast Cancer Cell Lines and Primary Breast Cancer Tissues: A Novel Role in Tumor Suppression?
Source: PLoS One. 2014 Jul 18;9(7):e102558. doi: 10.1371/journal.pone.0102558 (PMC4103839; doi:10.1371/journal.pone.0102558)
Supplement: Table S2 — Detailed gene list of the 87 gene signature of basal-A BT20 cells. (DOC) [file pone.0102558.s002.doc]

**Supporting Information S2**

**Table S2**. Detailed gene list of the 87 gene signature of basal-A BT20 cells.

| **Name** | **Symbol** | **ProbeSet** | **Fold-change** | **Parametric p-value** |
| --- | --- | --- | --- | --- |
|
| Secreted frizzled-related protein 1 | [SFRP1](http://www.ncbi.nlm.nih.gov/entrez/query.fcgi?cmd=search&db=gene&term=SFRP1) | [228413_s_at](https://www.affymetrix.com/LinkServlet?probeset=228413_s_at) | 224.33 | < 1e-07 |
| Kinesin family member 27 pseudogene | [LOC389765](http://www.ncbi.nlm.nih.gov/entrez/query.fcgi?cmd=search&db=gene&term=LOC389765) | [230815_at](https://www.affymetrix.com/LinkServlet?probeset=230815_at) | 0.5 | 0.0001234 |
| EF-hand calcium binding domain 7 | [EFCAB7](http://www.ncbi.nlm.nih.gov/entrez/query.fcgi?cmd=search&db=gene&term=EFCAB7) | [226800_at](https://www.affymetrix.com/LinkServlet?probeset=226800_at) | 0.49 | 0.0002011 |
| Protein-L-isoaspartate (D-aspartate) O-methyltransferase domain containing 1 | [PCMTD1](http://www.ncbi.nlm.nih.gov/entrez/query.fcgi?cmd=search&db=gene&term=PCMTD1) | [226119_at](https://www.affymetrix.com/LinkServlet?probeset=226119_at) | 0.48 | 0.000304 |
| Vav 3 guanine nucleotide exchange factor | [VAV3](http://www.ncbi.nlm.nih.gov/entrez/query.fcgi?cmd=search&db=gene&term=VAV3) | [218806_s_at](https://www.affymetrix.com/LinkServlet?probeset=218806_s_at) | 0.48 | 0.0003562 |
| Zinc finger protein 711 | [ZNF711](http://www.ncbi.nlm.nih.gov/entrez/query.fcgi?cmd=search&db=gene&term=ZNF711) | [228988_at](https://www.affymetrix.com/LinkServlet?probeset=228988_at) | 0.46 | 0.0003656 |
| Chitobiase, di-N-acetyl- | [CTBS](http://www.ncbi.nlm.nih.gov/entrez/query.fcgi?cmd=search&db=gene&term=CTBS) | [218923_at](https://www.affymetrix.com/LinkServlet?probeset=218923_at) | 0.35 | 0.0005222 |
| Methionyl-tRNA synthetase | [MARS](http://www.ncbi.nlm.nih.gov/entrez/query.fcgi?cmd=search&db=gene&term=MARS) | [213672_at](https://www.affymetrix.com/LinkServlet?probeset=213672_at) | 0.38 | 0.0007306 |
| Uncharacterized protein FLJ10038 | [FLJ10038](http://www.ncbi.nlm.nih.gov/entrez/query.fcgi?cmd=search&db=gene&term=FLJ10038) | [236164_at](https://www.affymetrix.com/LinkServlet?probeset=236164_at) | 0.29 | 0.0007378 |
| ZBED3 antisense RNA 1 | [ZBED3-AS1](http://www.ncbi.nlm.nih.gov/entrez/query.fcgi?cmd=search&db=gene&term=ZBED3-AS1) | [1564475_s_at](https://www.affymetrix.com/LinkServlet?probeset=1564475_s_at) | 0.49 | 0.0007851 |
| Zinc finger protein 165 | [ZNF165](http://www.ncbi.nlm.nih.gov/entrez/query.fcgi?cmd=search&db=gene&term=ZNF165) | [206683_at](https://www.affymetrix.com/LinkServlet?probeset=206683_at) | 0.5 | 0.0009163 |
| Zinc finger protein 25 | [ZNF25](http://www.ncbi.nlm.nih.gov/entrez/query.fcgi?cmd=search&db=gene&term=ZNF25) | [235164_at](https://www.affymetrix.com/LinkServlet?probeset=235164_at) | 0.48 | 0.0009645 |
| Branched chain amino-acid transaminase 1, cytosolic | [BCAT1](http://www.ncbi.nlm.nih.gov/entrez/query.fcgi?cmd=search&db=gene&term=BCAT1) | [226517_at](https://www.affymetrix.com/LinkServlet?probeset=226517_at) | 0.24 | 0.0017541 |
| Cysteine sulfinic acid decarboxylase | [CSAD](http://www.ncbi.nlm.nih.gov/entrez/query.fcgi?cmd=search&db=gene&term=CSAD) | [221139_s_at](https://www.affymetrix.com/LinkServlet?probeset=221139_s_at) | 0.38 | 0.0017778 |
| Rap guanine nucleotide exchange factor (GEF) 5 | [RAPGEF5](http://www.ncbi.nlm.nih.gov/entrez/query.fcgi?cmd=search&db=gene&term=RAPGEF5) | [204681_s_at](https://www.affymetrix.com/LinkServlet?probeset=204681_s_at) | 0.43 | 0.0020039 |
| LIM and cysteine-rich domains 1 | [LMCD1](http://www.ncbi.nlm.nih.gov/entrez/query.fcgi?cmd=search&db=gene&term=LMCD1) | [242767_at](https://www.affymetrix.com/LinkServlet?probeset=242767_at) | 2.71 | 0.0020053 |
| V-raf murine sarcoma 3611 viral oncogene homolog | [ARAF](http://www.ncbi.nlm.nih.gov/entrez/query.fcgi?cmd=search&db=gene&term=ARAF) | [230652_at](https://www.affymetrix.com/LinkServlet?probeset=230652_at) | 0.5 | 0.0021513 |
| Tissue factor pathway inhibitor 2 | [TFPI2](http://www.ncbi.nlm.nih.gov/entrez/query.fcgi?cmd=search&db=gene&term=TFPI2) | [209278_s_at](https://www.affymetrix.com/LinkServlet?probeset=209278_s_at) | 2.6 | 0.0027976 |
| Oxysterol binding protein-like 6 | [OSBPL6](http://www.ncbi.nlm.nih.gov/entrez/query.fcgi?cmd=search&db=gene&term=OSBPL6) | [236261_at](https://www.affymetrix.com/LinkServlet?probeset=236261_at) | 0.46 | 0.0033407 |
| Blocked early in transport 1 homolog (S. cerevisiae) | [BET1](http://www.ncbi.nlm.nih.gov/entrez/query.fcgi?cmd=search&db=gene&term=BET1) | [202710_at](https://www.affymetrix.com/LinkServlet?probeset=202710_at) | 0.47 | 0.0033584 |
| Four and a half LIM domains 2 | [FHL2](http://www.ncbi.nlm.nih.gov/entrez/query.fcgi?cmd=search&db=gene&term=FHL2) | [202949_s_at](https://www.affymetrix.com/LinkServlet?probeset=202949_s_at) | 2.52 | 0.0038468 |
| Transmembrane protein 55A | [TMEM55A](http://www.ncbi.nlm.nih.gov/entrez/query.fcgi?cmd=search&db=gene&term=TMEM55A) | [226338_at](https://www.affymetrix.com/LinkServlet?probeset=226338_at) | 0.49 | 0.0044466 |
| Histocompatibility (minor) HA-1 | [HMHA1](http://www.ncbi.nlm.nih.gov/entrez/query.fcgi?cmd=search&db=gene&term=HMHA1) | [212873_at](https://www.affymetrix.com/LinkServlet?probeset=212873_at) | 2.21 | 0.0051576 |
| Pleckstrin homology domain containing, family H (with MyTH4 domain) member 2 | [PLEKHH2](http://www.ncbi.nlm.nih.gov/entrez/query.fcgi?cmd=search&db=gene&term=PLEKHH2) | [227148_at](https://www.affymetrix.com/LinkServlet?probeset=227148_at) | 0.39 | 0.0051705 |
| Cortexin 1 | [CTXN1](http://www.ncbi.nlm.nih.gov/entrez/query.fcgi?cmd=search&db=gene&term=CTXN1) | [228126_x_at](https://www.affymetrix.com/LinkServlet?probeset=228126_x_at) | 2.02 | 0.0053264 |
| Chromosome 17 open reading frame 100 | [C17orf100](http://www.ncbi.nlm.nih.gov/entrez/query.fcgi?cmd=search&db=gene&term=C17orf100) | [229071_at](https://www.affymetrix.com/LinkServlet?probeset=229071_at) | 0.48 | 0.0054899 |
| Coiled-coil domain containing 91 | [CCDC91](http://www.ncbi.nlm.nih.gov/entrez/query.fcgi?cmd=search&db=gene&term=CCDC91) | [218545_at](https://www.affymetrix.com/LinkServlet?probeset=218545_at) | 0.47 | 0.0056954 |
| Junction mediating and regulatory protein, p53 cofactor | [JMY](http://www.ncbi.nlm.nih.gov/entrez/query.fcgi?cmd=search&db=gene&term=JMY) | [226352_at](https://www.affymetrix.com/LinkServlet?probeset=226352_at) | 0.47 | 0.0065612 |
| Solute carrier family 7 (anionic amino acid transporter light chain, xc- system), member 11 | [SLC7A11](http://www.ncbi.nlm.nih.gov/entrez/query.fcgi?cmd=search&db=gene&term=SLC7A11) | [217678_at](https://www.affymetrix.com/LinkServlet?probeset=217678_at) | 0.15 | 0.006872 |
| Uncharacterized LOC100287497 | [LOC100287497](http://www.ncbi.nlm.nih.gov/entrez/query.fcgi?cmd=search&db=gene&term=LOC100287497) | [234759_at](https://www.affymetrix.com/LinkServlet?probeset=234759_at) | 0.42 | 0.0074545 |
| Regulator of chromosome condensation (RCC1) and BTB (POZ) domain containing protein 1 | [RCBTB1](http://www.ncbi.nlm.nih.gov/entrez/query.fcgi?cmd=search&db=gene&term=RCBTB1) | [218352_at](https://www.affymetrix.com/LinkServlet?probeset=218352_at) | 0.41 | 0.0074782 |
| Uncharacterized LOC440905 | [LOC440905](http://www.ncbi.nlm.nih.gov/entrez/query.fcgi?cmd=search&db=gene&term=LOC440905) | [241895_at](https://www.affymetrix.com/LinkServlet?probeset=241895_at) | 0.46 | 0.0075147 |
| Amyloid beta (A4) precursor-like protein 1 | [APLP1](http://www.ncbi.nlm.nih.gov/entrez/query.fcgi?cmd=search&db=gene&term=APLP1) | [209462_at](https://www.affymetrix.com/LinkServlet?probeset=209462_at) | 2.16 | 0.0077206 |
| RB1-inducible coiled-coil 1 | [RB1CC1](http://www.ncbi.nlm.nih.gov/entrez/query.fcgi?cmd=search&db=gene&term=RB1CC1) | [202034_x_at](https://www.affymetrix.com/LinkServlet?probeset=202034_x_at) | 0.47 | 0.0080352 |
| Sterile alpha motif domain containing 4A | [SAMD4A](http://www.ncbi.nlm.nih.gov/entrez/query.fcgi?cmd=search&db=gene&term=SAMD4A) | [215495_s_at](https://www.affymetrix.com/LinkServlet?probeset=215495_s_at) | 2.24 | 0.0080893 |
| EPS8-like 1 | [EPS8L1](http://www.ncbi.nlm.nih.gov/entrez/query.fcgi?cmd=search&db=gene&term=EPS8L1) | [221665_s_at](https://www.affymetrix.com/LinkServlet?probeset=221665_s_at) | 2.02 | 0.008957 |
| E2F transcription factor 2 | [E2F2](http://www.ncbi.nlm.nih.gov/entrez/query.fcgi?cmd=search&db=gene&term=E2F2) | [207042_at](https://www.affymetrix.com/LinkServlet?probeset=207042_at) | 2.03 | 0.0100713 |
| S100 calcium binding protein P | [S100P](http://www.ncbi.nlm.nih.gov/entrez/query.fcgi?cmd=search&db=gene&term=S100P) | [204351_at](https://www.affymetrix.com/LinkServlet?probeset=204351_at) | 0.35 | 0.0100756 |
| Yippee-like 2 (Drosophila) | [YPEL2](http://www.ncbi.nlm.nih.gov/entrez/query.fcgi?cmd=search&db=gene&term=YPEL2) | [227020_at](https://www.affymetrix.com/LinkServlet?probeset=227020_at) | 0.45 | 0.0120317 |
| Chromosome 17 open reading frame 109 | [C17orf109](http://www.ncbi.nlm.nih.gov/entrez/query.fcgi?cmd=search&db=gene&term=C17orf109) | [229740_at](https://www.affymetrix.com/LinkServlet?probeset=229740_at) | 2.18 | 0.0127724 |
| Cytoplasmic polyadenylation element binding protein 3 | [CPEB3](http://www.ncbi.nlm.nih.gov/entrez/query.fcgi?cmd=search&db=gene&term=CPEB3) | [205773_at](https://www.affymetrix.com/LinkServlet?probeset=205773_at) | 0.39 | 0.0129404 |
| Interferon regulatory factor 2 binding protein 2 | [IRF2BP2](http://www.ncbi.nlm.nih.gov/entrez/query.fcgi?cmd=search&db=gene&term=IRF2BP2) | [224571_at](https://www.affymetrix.com/LinkServlet?probeset=224571_at) | 0.5 | 0.0136015 |
| SUN domain containing ossification factor | [SUCO](http://www.ncbi.nlm.nih.gov/entrez/query.fcgi?cmd=search&db=gene&term=SUCO) | [203429_s_at](https://www.affymetrix.com/LinkServlet?probeset=203429_s_at) | 0.49 | 0.0137316 |
| Zinc finger and SCAN domain containing 2 | [ZSCAN2](http://www.ncbi.nlm.nih.gov/entrez/query.fcgi?cmd=search&db=gene&term=ZSCAN2) | [231188_at](https://www.affymetrix.com/LinkServlet?probeset=231188_at) | 0.45 | 0.0140703 |
| Inverted formin, FH2 and WH2 domain containing | [INF2](http://www.ncbi.nlm.nih.gov/entrez/query.fcgi?cmd=search&db=gene&term=INF2) | [224469_s_at](https://www.affymetrix.com/LinkServlet?probeset=224469_s_at) | 2.5 | 0.014083 |
| Family with sequence similarity 149, member A | [FAM149A](http://www.ncbi.nlm.nih.gov/entrez/query.fcgi?cmd=search&db=gene&term=FAM149A) | [214889_at](https://www.affymetrix.com/LinkServlet?probeset=214889_at) | 2.07 | 0.0148017 |
| Ribosomal protein L31 | [RPL31](http://www.ncbi.nlm.nih.gov/entrez/query.fcgi?cmd=search&db=gene&term=RPL31) | [200962_at](https://www.affymetrix.com/LinkServlet?probeset=200962_at) | 0.48 | 0.0153843 |
| DnaJ (Hsp40) homolog, subfamily B, member 9 | [DNAJB9](http://www.ncbi.nlm.nih.gov/entrez/query.fcgi?cmd=search&db=gene&term=DNAJB9) | [202843_at](https://www.affymetrix.com/LinkServlet?probeset=202843_at) | 0.31 | 0.0158096 |
| H19, imprinted maternally expressed transcript (non-protein coding) | [H19](http://www.ncbi.nlm.nih.gov/entrez/query.fcgi?cmd=search&db=gene&term=H19) | [224646_x_at](https://www.affymetrix.com/LinkServlet?probeset=224646_x_at) | 2.03 | 0.0166771 |
| Pyridine nucleotide-disulphide oxidoreductase domain 1 | [PYROXD1](http://www.ncbi.nlm.nih.gov/entrez/query.fcgi?cmd=search&db=gene&term=PYROXD1) | [213878_at](https://www.affymetrix.com/LinkServlet?probeset=213878_at) | 0.46 | 0.0178515 |
| Solute carrier family 30 (zinc transporter), member 7 | [SLC30A7](http://www.ncbi.nlm.nih.gov/entrez/query.fcgi?cmd=search&db=gene&term=SLC30A7) | [226601_at](https://www.affymetrix.com/LinkServlet?probeset=226601_at) | 0.43 | 0.0189389 |
| Uncharacterized LOC399717 | [FLJ45983](http://www.ncbi.nlm.nih.gov/entrez/query.fcgi?cmd=search&db=gene&term=FLJ45983) | [240827_at](https://www.affymetrix.com/LinkServlet?probeset=240827_at) | 2.38 | 0.0196574 |
| Leucine zipper transcription factor-like 1 | [LZTFL1](http://www.ncbi.nlm.nih.gov/entrez/query.fcgi?cmd=search&db=gene&term=LZTFL1) | [218437_s_at](https://www.affymetrix.com/LinkServlet?probeset=218437_s_at) | 0.33 | 0.0200931 |
| Ubiquitin specific peptidase 8 | [USP8](http://www.ncbi.nlm.nih.gov/entrez/query.fcgi?cmd=search&db=gene&term=USP8) | [229501_s_at](https://www.affymetrix.com/LinkServlet?probeset=229501_s_at) | 0.33 | 0.0220139 |
| Proline-rich nuclear receptor coactivator 1 | [PNRC1](http://www.ncbi.nlm.nih.gov/entrez/query.fcgi?cmd=search&db=gene&term=PNRC1) | [209034_at](https://www.affymetrix.com/LinkServlet?probeset=209034_at) | 0.39 | 0.0228968 |
| Eukaryotic translation elongation factor 1 alpha 2 | [EEF1A2](http://www.ncbi.nlm.nih.gov/entrez/query.fcgi?cmd=search&db=gene&term=EEF1A2) | [204540_at](https://www.affymetrix.com/LinkServlet?probeset=204540_at) | 2.07 | 0.0239374 |
| ERO1-like beta (S. cerevisiae) | [ERO1LB](http://www.ncbi.nlm.nih.gov/entrez/query.fcgi?cmd=search&db=gene&term=ERO1LB) | [231944_at](https://www.affymetrix.com/LinkServlet?probeset=231944_at) | 0.45 | 0.0240959 |
| Homocysteine-inducible, endoplasmic reticulum stress-inducible, ubiquitin-like domain member 1 | [HERPUD1](http://www.ncbi.nlm.nih.gov/entrez/query.fcgi?cmd=search&db=gene&term=HERPUD1) | [217168_s_at](https://www.affymetrix.com/LinkServlet?probeset=217168_s_at) | 0.37 | 0.0250378 |
| Forkhead box P2 | [FOXP2](http://www.ncbi.nlm.nih.gov/entrez/query.fcgi?cmd=search&db=gene&term=FOXP2) | [243278_at](https://www.affymetrix.com/LinkServlet?probeset=243278_at) | 0.48 | 0.0252233 |
| Gliomedin | [GLDN](http://www.ncbi.nlm.nih.gov/entrez/query.fcgi?cmd=search&db=gene&term=GLDN) | [230360_at](https://www.affymetrix.com/LinkServlet?probeset=230360_at) | 0.44 | 0.0255275 |
| Transmembrane protein 45A | [TMEM45A](http://www.ncbi.nlm.nih.gov/entrez/query.fcgi?cmd=search&db=gene&term=TMEM45A) | [219410_at](https://www.affymetrix.com/LinkServlet?probeset=219410_at) | 0.35 | 0.0258597 |
| GABPB1 antisense RNA 1 | [GABPB1-AS1](http://www.ncbi.nlm.nih.gov/entrez/query.fcgi?cmd=search&db=gene&term=GABPB1-AS1) | [227406_at](https://www.affymetrix.com/LinkServlet?probeset=227406_at) | 0.4 | 0.0258728 |
| Small glutamine-rich tetratricopeptide repeat (TPR)-containing, beta | [SGTB](http://www.ncbi.nlm.nih.gov/entrez/query.fcgi?cmd=search&db=gene&term=SGTB) | [228745_at](https://www.affymetrix.com/LinkServlet?probeset=228745_at) | 0.37 | 0.0258934 |
| Uncharacterized LOC145788 | [FLJ27352](http://www.ncbi.nlm.nih.gov/entrez/query.fcgi?cmd=search&db=gene&term=FLJ27352) | [243309_at](https://www.affymetrix.com/LinkServlet?probeset=243309_at) | 0.37 | 0.0263085 |
| Transmembrane and coiled-coil domains 3 | [TMCO3](http://www.ncbi.nlm.nih.gov/entrez/query.fcgi?cmd=search&db=gene&term=TMCO3) | [220241_at](https://www.affymetrix.com/LinkServlet?probeset=220241_at) | 0.44 | 0.0272334 |
| Membrane associated guanylate kinase, WW and PDZ domain containing 2 | [MAGI2](http://www.ncbi.nlm.nih.gov/entrez/query.fcgi?cmd=search&db=gene&term=MAGI2) | [209737_at](https://www.affymetrix.com/LinkServlet?probeset=209737_at) | 0.47 | 0.0273175 |
| Microtubule-associated protein 2 | [MAP2](http://www.ncbi.nlm.nih.gov/entrez/query.fcgi?cmd=search&db=gene&term=MAP2) | [225540_at](https://www.affymetrix.com/LinkServlet?probeset=225540_at) | 0.39 | 0.0274736 |
| Matrix-remodelling associated 7 | [MXRA7](http://www.ncbi.nlm.nih.gov/entrez/query.fcgi?cmd=search&db=gene&term=MXRA7) | [212509_s_at](https://www.affymetrix.com/LinkServlet?probeset=212509_s_at) | 2.19 | 0.028706 |
| SEC24 family, member D (S. cerevisiae) | [SEC24D](http://www.ncbi.nlm.nih.gov/entrez/query.fcgi?cmd=search&db=gene&term=SEC24D) | [202375_at](https://www.affymetrix.com/LinkServlet?probeset=202375_at) | 0.48 | 0.0287636 |
| ERGIC and golgi 2 | [ERGIC2](http://www.ncbi.nlm.nih.gov/entrez/query.fcgi?cmd=search&db=gene&term=ERGIC2) | [226422_at](https://www.affymetrix.com/LinkServlet?probeset=226422_at) | 0.44 | 0.0292216 |
| EGF containing fibulin-like extracellular matrix protein 1 | [EFEMP1](http://www.ncbi.nlm.nih.gov/entrez/query.fcgi?cmd=search&db=gene&term=EFEMP1) | [201842_s_at](https://www.affymetrix.com/LinkServlet?probeset=201842_s_at) | 2.6 | 0.0308642 |
| Family with sequence similarity 214, member A | [FAM214A](http://www.ncbi.nlm.nih.gov/entrez/query.fcgi?cmd=search&db=gene&term=FAM214A) | [225327_at](https://www.affymetrix.com/LinkServlet?probeset=225327_at) | 0.47 | 0.0320071 |
| Proline rich 15 | [PRR15](http://www.ncbi.nlm.nih.gov/entrez/query.fcgi?cmd=search&db=gene&term=PRR15) | [226961_at](https://www.affymetrix.com/LinkServlet?probeset=226961_at) | 0.42 | 0.0325794 |
| Polymerase (DNA directed), alpha 2, accessory subunit | [POLA2](http://www.ncbi.nlm.nih.gov/entrez/query.fcgi?cmd=search&db=gene&term=POLA2) | [204441_s_at](https://www.affymetrix.com/LinkServlet?probeset=204441_s_at) | 2.19 | 0.0346284 |
| Transforming growth factor, beta-induced, 68kDa | [TGFBI](http://www.ncbi.nlm.nih.gov/entrez/query.fcgi?cmd=search&db=gene&term=TGFBI) | [201506_at](https://www.affymetrix.com/LinkServlet?probeset=201506_at) | 0.45 | 0.0347521 |
| SR-related CTD-associated factor 11 | [SCAF11](http://www.ncbi.nlm.nih.gov/entrez/query.fcgi?cmd=search&db=gene&term=SCAF11) | [235579_at](https://www.affymetrix.com/LinkServlet?probeset=235579_at) | 3.42 | 0.036097 |
| Angiogenin, ribonuclease, RNase A family, 5 | [ANG](http://www.ncbi.nlm.nih.gov/entrez/query.fcgi?cmd=search&db=gene&term=ANG) | [205141_at](https://www.affymetrix.com/LinkServlet?probeset=205141_at) | 0.29 | 0.0366423 |
| Laminin, beta 1 | [LAMB1](http://www.ncbi.nlm.nih.gov/entrez/query.fcgi?cmd=search&db=gene&term=LAMB1) | [201505_at](https://www.affymetrix.com/LinkServlet?probeset=201505_at) | 2.65 | 0.0380295 |
| Ras homolog family member Q | [RHOQ](http://www.ncbi.nlm.nih.gov/entrez/query.fcgi?cmd=search&db=gene&term=RHOQ) | [212119_at](https://www.affymetrix.com/LinkServlet?probeset=212119_at) | 0.48 | 0.0405056 |
| Aldehyde dehydrogenase 1 family, member L2 | [ALDH1L2](http://www.ncbi.nlm.nih.gov/entrez/query.fcgi?cmd=search&db=gene&term=ALDH1L2) | [231202_at](https://www.affymetrix.com/LinkServlet?probeset=231202_at) | 0.24 | 0.0407878 |
| Brain expressed X-linked 2 | [BEX2](http://www.ncbi.nlm.nih.gov/entrez/query.fcgi?cmd=search&db=gene&term=BEX2) | [224367_at](https://www.affymetrix.com/LinkServlet?probeset=224367_at) | 0.23 | 0.0417787 |
| DNA-damage-inducible transcript 4 | [DDIT4](http://www.ncbi.nlm.nih.gov/entrez/query.fcgi?cmd=search&db=gene&term=DDIT4) | [202887_s_at](https://www.affymetrix.com/LinkServlet?probeset=202887_s_at) | 0.2 | 0.0423147 |
| Cell cycle progression 1 | [CCPG1](http://www.ncbi.nlm.nih.gov/entrez/query.fcgi?cmd=search&db=gene&term=CCPG1) | [222156_x_at](https://www.affymetrix.com/LinkServlet?probeset=222156_x_at) | 0.49 | 0.0437862 |
| Furry homolog (Drosophila) | [FRY](http://www.ncbi.nlm.nih.gov/entrez/query.fcgi?cmd=search&db=gene&term=FRY) | [204072_s_at](https://www.affymetrix.com/LinkServlet?probeset=204072_s_at) | 0.43 | 0.0452643 |
| Tubulin, epsilon 1 | [TUBE1](http://www.ncbi.nlm.nih.gov/entrez/query.fcgi?cmd=search&db=gene&term=TUBE1) | [226181_at](https://www.affymetrix.com/LinkServlet?probeset=226181_at) | 0.38 | 0.046189 |
| Uncharacterized LOC100289092 | [LOC100289092](http://www.ncbi.nlm.nih.gov/entrez/query.fcgi?cmd=search&db=gene&term=LOC100289092) | [230696_at](https://www.affymetrix.com/LinkServlet?probeset=230696_at) | 2.07 | 0.0486829 |
| Calcitonin receptor | [CALCR](http://www.ncbi.nlm.nih.gov/entrez/query.fcgi?cmd=search&db=gene&term=CALCR) | [207886_s_at](https://www.affymetrix.com/LinkServlet?probeset=207886_s_at) | 2.79 | 0.0494232 |
