# Supplementary material for: BDNF Is Associated with SFRP1 Expression in Luminal and Basal-Like Breast Cancer Cell Lines and Primary Breast Cancer Tissues: A Novel Role in Tumor Suppression?
Source: PLoS One. 2014 Jul 18;9(7):e102558. doi: 10.1371/journal.pone.0102558 (PMC4103839; doi:10.1371/journal.pone.0102558)
Supplement: Table S3 — Detailed gene list of the 104 gene signature of luminal SKBR3 cells. (DOC) [file pone.0102558.s003.doc]

**Supporting Information S3**

**Table S3. Detailed gene list of the 104 gene signature of luminal SKBR3 cells.**

| **Name** | **Symbol** | **ProbeSet** | **Fold-change** | **Parametric p-value** |
| --- | --- | --- | --- | --- |
|
| Secreted frizzled-related protein 1 | [SFRP1](http://www.ncbi.nlm.nih.gov/entrez/query.fcgi?cmd=search&db=gene&term=SFRP1) | [228413_s_at](https://www.affymetrix.com/LinkServlet?probeset=228413_s_at) | 344.33 | < 1e-07 |
| Inhibin, beta A | [INHBA](http://www.ncbi.nlm.nih.gov/entrez/query.fcgi?cmd=search&db=gene&term=INHBA) | [204926_at](https://www.affymetrix.com/LinkServlet?probeset=204926_at) | 0.18 | 6,00E-06 |
| Aldo-keto reductase family 1, member C2 (dihydrodiol dehydrogenase 2; bile acid binding protein; 3-alpha hydroxysteroid dehydrogenase, type III) | [AKR1C2](http://www.ncbi.nlm.nih.gov/entrez/query.fcgi?cmd=search&db=gene&term=AKR1C2) | [211653_x_at](https://www.affymetrix.com/LinkServlet?probeset=211653_x_at) | 0.26 | 9.2e-06 |
| Aldo-keto reductase family 1, member C1 (dihydrodiol dehydrogenase 1; 20-alpha (3-alpha)-hydroxysteroid dehydrogenase) | [AKR1C1](http://www.ncbi.nlm.nih.gov/entrez/query.fcgi?cmd=search&db=gene&term=AKR1C1) | [204151_x_at](https://www.affymetrix.com/LinkServlet?probeset=204151_x_at) | 0.21 | 2.88e-05 |
| Chromosome 11 open reading frame 96 | [C11orf96](http://www.ncbi.nlm.nih.gov/entrez/query.fcgi?cmd=search&db=gene&term=C11orf96) | [227099_s_at](https://www.affymetrix.com/LinkServlet?probeset=227099_s_at) | 0.11 | 4.6e-05 |
| Matrix metallopeptidase 3 (stromelysin 1, progelatinase) | [MMP3](http://www.ncbi.nlm.nih.gov/entrez/query.fcgi?cmd=search&db=gene&term=MMP3) | [205828_at](https://www.affymetrix.com/LinkServlet?probeset=205828_at) | 0.28 | 4.66e-05 |
| TNFAIP3 interacting protein 1 | [TNIP1](http://www.ncbi.nlm.nih.gov/entrez/query.fcgi?cmd=search&db=gene&term=TNIP1) | [243423_at](https://www.affymetrix.com/LinkServlet?probeset=243423_at) | 0.3 | 5.42e-05 |
| Matrix metallopeptidase 1 (interstitial collagenase) | [MMP1](http://www.ncbi.nlm.nih.gov/entrez/query.fcgi?cmd=search&db=gene&term=MMP1) | [204475_at](https://www.affymetrix.com/LinkServlet?probeset=204475_at) | 0.056 | 6.46e-05 |
| Spermidine/spermine N1-acetyltransferase 1 | [SAT1](http://www.ncbi.nlm.nih.gov/entrez/query.fcgi?cmd=search&db=gene&term=SAT1) | [213988_s_at](https://www.affymetrix.com/LinkServlet?probeset=213988_s_at) | 0.2 | 7.27e-05 |
| Integrin, alpha 2 (CD49B, alpha 2 subunit of VLA-2 receptor) | [ITGA2](http://www.ncbi.nlm.nih.gov/entrez/query.fcgi?cmd=search&db=gene&term=ITGA2) | [205032_at](https://www.affymetrix.com/LinkServlet?probeset=205032_at) | 0.21 | 0.0001626 |
| Lymphocyte antigen 96 | [LY96](http://www.ncbi.nlm.nih.gov/entrez/query.fcgi?cmd=search&db=gene&term=LY96) | [206584_at](https://www.affymetrix.com/LinkServlet?probeset=206584_at) | 0.15 | 0.0001795 |
| Hairy/enhancer-of-split related with YRPW motif 1 | [HEY1](http://www.ncbi.nlm.nih.gov/entrez/query.fcgi?cmd=search&db=gene&term=HEY1) | [44783_s_at](https://www.affymetrix.com/LinkServlet?probeset=44783_s_at) | 0.36 | 0.0001804 |
| Prostaglandin-endoperoxide synthase 2 (prostaglandin G/H synthase and cyclooxygenase) | [PTGS2](http://www.ncbi.nlm.nih.gov/entrez/query.fcgi?cmd=search&db=gene&term=PTGS2) | [1554997_a_at](https://www.affymetrix.com/LinkServlet?probeset=1554997_a_at) | 0.084 | 0.0002108 |
| Regulator of G-protein signaling 7 | [RGS7](http://www.ncbi.nlm.nih.gov/entrez/query.fcgi?cmd=search&db=gene&term=RGS7) | [206290_s_at](https://www.affymetrix.com/LinkServlet?probeset=206290_s_at) | 3.14 | 0.0002248 |
| Tripartite motif family-like 2 | [TRIML2](http://www.ncbi.nlm.nih.gov/entrez/query.fcgi?cmd=search&db=gene&term=TRIML2) | [1552580_at](https://www.affymetrix.com/LinkServlet?probeset=1552580_at) | 0.14 | 0.0003148 |
| S100 calcium binding protein A6 | [S100A6](http://www.ncbi.nlm.nih.gov/entrez/query.fcgi?cmd=search&db=gene&term=S100A6) | [228923_at](https://www.affymetrix.com/LinkServlet?probeset=228923_at) | 0.3 | 0.0003838 |
| Cbl proto-oncogene, E3 ubiquitin protein ligase B | [CBLB](http://www.ncbi.nlm.nih.gov/entrez/query.fcgi?cmd=search&db=gene&term=CBLB) | [209682_at](https://www.affymetrix.com/LinkServlet?probeset=209682_at) | 0.24 | 0.0004068 |
| Jun B proto-oncogene | [JUNB](http://www.ncbi.nlm.nih.gov/entrez/query.fcgi?cmd=search&db=gene&term=JUNB) | [201473_at](https://www.affymetrix.com/LinkServlet?probeset=201473_at) | 0.33 | 0.0004269 |
| Nicotinamide phosphoribosyltransferase | [NAMPT](http://www.ncbi.nlm.nih.gov/entrez/query.fcgi?cmd=search&db=gene&term=NAMPT) | [243296_at](https://www.affymetrix.com/LinkServlet?probeset=243296_at) | 0.2 | 0.0004325 |
| Brain-derived neurotrophic factor | [BDNF](http://www.ncbi.nlm.nih.gov/entrez/query.fcgi?cmd=search&db=gene&term=BDNF) | [239367_at](https://www.affymetrix.com/LinkServlet?probeset=239367_at) | 3.84 | 0.0005098 |
| Cyclin-dependent kinase inhibitor 1C (p57, Kip2) | [CDKN1C](http://www.ncbi.nlm.nih.gov/entrez/query.fcgi?cmd=search&db=gene&term=CDKN1C) | [213348_at](https://www.affymetrix.com/LinkServlet?probeset=213348_at) | 0.33 | 0.0006175 |
| Nuclear receptor subfamily 4, group A, member 2 | [NR4A2](http://www.ncbi.nlm.nih.gov/entrez/query.fcgi?cmd=search&db=gene&term=NR4A2) | [216248_s_at](https://www.affymetrix.com/LinkServlet?probeset=216248_s_at) | 0.29 | 0.0007196 |
| Lysyl oxidase | [LOX](http://www.ncbi.nlm.nih.gov/entrez/query.fcgi?cmd=search&db=gene&term=LOX) | [204298_s_at](https://www.affymetrix.com/LinkServlet?probeset=204298_s_at) | 0.29 | 0.0007513 |
| Stanniocalcin 1 | [STC1](http://www.ncbi.nlm.nih.gov/entrez/query.fcgi?cmd=search&db=gene&term=STC1) | [230746_s_at](https://www.affymetrix.com/LinkServlet?probeset=230746_s_at) | 0.073 | 0.0007654 |
| MOB kinase activator 3B | [MOB3B](http://www.ncbi.nlm.nih.gov/entrez/query.fcgi?cmd=search&db=gene&term=MOB3B) | [226844_at](https://www.affymetrix.com/LinkServlet?probeset=226844_at) | 0.34 | 0.0009019 |
| FBJ murine osteosarcoma viral oncogene homolog | [FOS](http://www.ncbi.nlm.nih.gov/entrez/query.fcgi?cmd=search&db=gene&term=FOS) | [209189_at](https://www.affymetrix.com/LinkServlet?probeset=209189_at) | 0.15 | 0.0009642 |
| Frizzled family receptor 8 | [FZD8](http://www.ncbi.nlm.nih.gov/entrez/query.fcgi?cmd=search&db=gene&term=FZD8) | [224325_at](https://www.affymetrix.com/LinkServlet?probeset=224325_at) | 0.37 | 0.0010946 |
| Solute carrier family 2 (facilitated glucose transporter), member 3 | [SLC2A3](http://www.ncbi.nlm.nih.gov/entrez/query.fcgi?cmd=search&db=gene&term=SLC2A3) | [202499_s_at](https://www.affymetrix.com/LinkServlet?probeset=202499_s_at) | 0.34 | 0.0010958 |
| ST3 beta-galactoside alpha-2,3-sialyltransferase 6 | [ST3GAL6](http://www.ncbi.nlm.nih.gov/entrez/query.fcgi?cmd=search&db=gene&term=ST3GAL6) | [213355_at](https://www.affymetrix.com/LinkServlet?probeset=213355_at) | 0.31 | 0.001106 |
| Asparagine-linked glycosylation 13 homolog (S. cerevisiae) | [ALG13](http://www.ncbi.nlm.nih.gov/entrez/query.fcgi?cmd=search&db=gene&term=ALG13) | [222808_at](https://www.affymetrix.com/LinkServlet?probeset=222808_at) | 2.36 | 0.0012679 |
| Angiogenin, ribonuclease, RNase A family, 5 | [ANG](http://www.ncbi.nlm.nih.gov/entrez/query.fcgi?cmd=search&db=gene&term=ANG) | [205141_at](https://www.affymetrix.com/LinkServlet?probeset=205141_at) | 0.3 | 0.0013688 |
| Keratin 15 | [KRT15](http://www.ncbi.nlm.nih.gov/entrez/query.fcgi?cmd=search&db=gene&term=KRT15) | [204734_at](https://www.affymetrix.com/LinkServlet?probeset=204734_at) | 0.36 | 0.0014102 |
| Chromosome 4 open reading frame 34 | [C4orf34](http://www.ncbi.nlm.nih.gov/entrez/query.fcgi?cmd=search&db=gene&term=C4orf34) | [224990_at](https://www.affymetrix.com/LinkServlet?probeset=224990_at) | 0.19 | 0.001463 |
| GTP binding protein overexpressed in skeletal muscle | [GEM](http://www.ncbi.nlm.nih.gov/entrez/query.fcgi?cmd=search&db=gene&term=GEM) | [204472_at](https://www.affymetrix.com/LinkServlet?probeset=204472_at) | 0.18 | 0.0014778 |
| Mesoderm specific transcript homolog (mouse) | [MEST](http://www.ncbi.nlm.nih.gov/entrez/query.fcgi?cmd=search&db=gene&term=MEST) | [202016_at](https://www.affymetrix.com/LinkServlet?probeset=202016_at) | 2.79 | 0.0016085 |
| ChaC, cation transport regulator homolog 2 (E. coli) | [CHAC2](http://www.ncbi.nlm.nih.gov/entrez/query.fcgi?cmd=search&db=gene&term=CHAC2) | [235117_at](https://www.affymetrix.com/LinkServlet?probeset=235117_at) | 2.37 | 0.001638 |
| RAS, dexamethasone-induced 1 | [RASD1](http://www.ncbi.nlm.nih.gov/entrez/query.fcgi?cmd=search&db=gene&term=RASD1) | [223467_at](https://www.affymetrix.com/LinkServlet?probeset=223467_at) | 0.18 | 0.0018138 |
| Solute carrier family 9, subfamily A (NHE7, cation proton antiporter 7), member 7 | [SLC9A7](http://www.ncbi.nlm.nih.gov/entrez/query.fcgi?cmd=search&db=gene&term=SLC9A7) | [1552671_a_at](https://www.affymetrix.com/LinkServlet?probeset=1552671_a_at) | 0.44 | 0.0019286 |
| SRY (sex determining region Y)-box 4 | [SOX4](http://www.ncbi.nlm.nih.gov/entrez/query.fcgi?cmd=search&db=gene&term=SOX4) | [201416_at](https://www.affymetrix.com/LinkServlet?probeset=201416_at) | 0.26 | 0.0020045 |
| B9 protein domain 1 | [B9D1](http://www.ncbi.nlm.nih.gov/entrez/query.fcgi?cmd=search&db=gene&term=B9D1) | [210534_s_at](https://www.affymetrix.com/LinkServlet?probeset=210534_s_at) | 0.4 | 0.0020428 |
| Colony stimulating factor 2 (granulocyte-macrophage) | [CSF2](http://www.ncbi.nlm.nih.gov/entrez/query.fcgi?cmd=search&db=gene&term=CSF2) | [210229_s_at](https://www.affymetrix.com/LinkServlet?probeset=210229_s_at) | 0.14 | 0.0021136 |
| Chloride channel, voltage-sensitive 4 | [CLCN4](http://www.ncbi.nlm.nih.gov/entrez/query.fcgi?cmd=search&db=gene&term=CLCN4) | [214769_at](https://www.affymetrix.com/LinkServlet?probeset=214769_at) | 2.64 | 0.0021929 |
| serpin peptidase inhibitor, clade D (heparin cofactor), member 1 | [SERPIND1](http://www.ncbi.nlm.nih.gov/entrez/query.fcgi?cmd=search&db=gene&term=SERPIND1) | [205576_at](https://www.affymetrix.com/LinkServlet?probeset=205576_at) | 0.34 | 0.0023385 |
| MATN1 antisense RNA 1 | [MATN1-AS1](http://www.ncbi.nlm.nih.gov/entrez/query.fcgi?cmd=search&db=gene&term=MATN1-AS1) | [1557558_s_at](https://www.affymetrix.com/LinkServlet?probeset=1557558_s_at) | 0.34 | 0.0024109 |
| Basic helix-loop-helix family, member e40 | [BHLHE40](http://www.ncbi.nlm.nih.gov/entrez/query.fcgi?cmd=search&db=gene&term=BHLHE40) | [201170_s_at](https://www.affymetrix.com/LinkServlet?probeset=201170_s_at) | 0.19 | 0.0027046 |
| Hepatocyte nuclear factor 4, gamma | [HNF4G](http://www.ncbi.nlm.nih.gov/entrez/query.fcgi?cmd=search&db=gene&term=HNF4G) | [232271_at](https://www.affymetrix.com/LinkServlet?probeset=232271_at) | 0.4 | 0.0029124 |
| Methionyl-tRNA synthetase 2, mitochondrial | [MARS2](http://www.ncbi.nlm.nih.gov/entrez/query.fcgi?cmd=search&db=gene&term=MARS2) | [243529_at](https://www.affymetrix.com/LinkServlet?probeset=243529_at) | 2.27 | 0.0031617 |
| Regulator of G-protein signaling 17 | [RGS17](http://www.ncbi.nlm.nih.gov/entrez/query.fcgi?cmd=search&db=gene&term=RGS17) | [220334_at](https://www.affymetrix.com/LinkServlet?probeset=220334_at) | 0.37 | 0.0035768 |
| Nuclear factor of kappa light polypeptide gene enhancer in B-cells inhibitor, alpha | [NFKBIA](http://www.ncbi.nlm.nih.gov/entrez/query.fcgi?cmd=search&db=gene&term=NFKBIA) | [201502_s_at](https://www.affymetrix.com/LinkServlet?probeset=201502_s_at) | 0.27 | 0.0035876 |
| Nuclear factor of kappa light polypeptide gene enhancer in B-cells inhibitor, zeta | [NFKBIZ](http://www.ncbi.nlm.nih.gov/entrez/query.fcgi?cmd=search&db=gene&term=NFKBIZ) | [223217_s_at](https://www.affymetrix.com/LinkServlet?probeset=223217_s_at) | 0.21 | 0.0035933 |
| Nuclear receptor subfamily 4, group A, member 1 | [NR4A1](http://www.ncbi.nlm.nih.gov/entrez/query.fcgi?cmd=search&db=gene&term=NR4A1) | [202340_x_at](https://www.affymetrix.com/LinkServlet?probeset=202340_x_at) | 0.39 | 0.0036466 |
| HECT, C2 and WW domain containing E3 ubiquitin protein ligase 2 | [HECW2](http://www.ncbi.nlm.nih.gov/entrez/query.fcgi?cmd=search&db=gene&term=HECW2) | [232080_at](https://www.affymetrix.com/LinkServlet?probeset=232080_at) | 0.35 | 0.0039374 |
| Meiosis inhibitor 1 | [MEI1](http://www.ncbi.nlm.nih.gov/entrez/query.fcgi?cmd=search&db=gene&term=MEI1) | [230011_at](https://www.affymetrix.com/LinkServlet?probeset=230011_at) | 0.39 | 0.0039519 |
| C-type lectin domain family 11, member A | [CLEC11A](http://www.ncbi.nlm.nih.gov/entrez/query.fcgi?cmd=search&db=gene&term=CLEC11A) | [211709_s_at](https://www.affymetrix.com/LinkServlet?probeset=211709_s_at) | 0.25 | 0.0041447 |
| Jumonji C domain containing histone demethylase 1 homolog D (S. cerevisiae) | [JHDM1D](http://www.ncbi.nlm.nih.gov/entrez/query.fcgi?cmd=search&db=gene&term=JHDM1D) | [221778_at](https://www.affymetrix.com/LinkServlet?probeset=221778_at) | 0.27 | 0.0041659 |
| Dual specificity phosphatase 16 | [DUSP16](http://www.ncbi.nlm.nih.gov/entrez/query.fcgi?cmd=search&db=gene&term=DUSP16) | [224832_at](https://www.affymetrix.com/LinkServlet?probeset=224832_at) | 0.26 | 0.0042309 |
| Tribbles homolog 1 (Drosophila) | [TRIB1](http://www.ncbi.nlm.nih.gov/entrez/query.fcgi?cmd=search&db=gene&term=TRIB1) | [202241_at](https://www.affymetrix.com/LinkServlet?probeset=202241_at) | 0.31 | 0.0042455 |
| Arrestin domain containing 3 | [ARRDC3](http://www.ncbi.nlm.nih.gov/entrez/query.fcgi?cmd=search&db=gene&term=ARRDC3) | [224797_at](https://www.affymetrix.com/LinkServlet?probeset=224797_at) | 0.39 | 0.0044333 |
| Ubiquitin-like with PHD and ring finger domains 1 | [UHRF1](http://www.ncbi.nlm.nih.gov/entrez/query.fcgi?cmd=search&db=gene&term=UHRF1) | [225655_at](https://www.affymetrix.com/LinkServlet?probeset=225655_at) | 2.70 | 0.0044492 |
| Cytochrome b5 reductase 2 | [CYB5R2](http://www.ncbi.nlm.nih.gov/entrez/query.fcgi?cmd=search&db=gene&term=CYB5R2) | [220230_s_at](https://www.affymetrix.com/LinkServlet?probeset=220230_s_at) | 0.4 | 0.0044495 |
| E2F transcription factor 8 | [E2F8](http://www.ncbi.nlm.nih.gov/entrez/query.fcgi?cmd=search&db=gene&term=E2F8) | [219990_at](https://www.affymetrix.com/LinkServlet?probeset=219990_at) | 2.54 | 0.0044551 |
| Immediate early response 3 | [IER3](http://www.ncbi.nlm.nih.gov/entrez/query.fcgi?cmd=search&db=gene&term=IER3) | [201631_s_at](https://www.affymetrix.com/LinkServlet?probeset=201631_s_at) | 0.27 | 0.0045022 |
| Phosphoprotein associated with glycosphingolipid microdomains 1 | [PAG1](http://www.ncbi.nlm.nih.gov/entrez/query.fcgi?cmd=search&db=gene&term=PAG1) | [225626_at](https://www.affymetrix.com/LinkServlet?probeset=225626_at) | 0.29 | 0.004555 |
| SERTA domain containing 4 | [SERTAD4](http://www.ncbi.nlm.nih.gov/entrez/query.fcgi?cmd=search&db=gene&term=SERTAD4) | [235337_at](https://www.affymetrix.com/LinkServlet?probeset=235337_at) | 3.1 | 0.0046638 |
| Interferon stimulated exonuclease gene 20kDa | [ISG20](http://www.ncbi.nlm.nih.gov/entrez/query.fcgi?cmd=search&db=gene&term=ISG20) | [204698_at](https://www.affymetrix.com/LinkServlet?probeset=204698_at) | 0.13 | 0.0047745 |
| G patch domain containing 4 | [GPATCH4](http://www.ncbi.nlm.nih.gov/entrez/query.fcgi?cmd=search&db=gene&term=GPATCH4) | [224634_at](https://www.affymetrix.com/LinkServlet?probeset=224634_at) | 2.23 | 0.0050395 |
| Sphingosine-1-phosphate receptor 1 | [S1PR1](http://www.ncbi.nlm.nih.gov/entrez/query.fcgi?cmd=search&db=gene&term=S1PR1) | [204642_at](https://www.affymetrix.com/LinkServlet?probeset=204642_at) | 2.92 | 0.0053074 |
| Fatty acid binding protein 4, adipocyte | [FABP4](http://www.ncbi.nlm.nih.gov/entrez/query.fcgi?cmd=search&db=gene&term=FABP4) | [203980_at](https://www.affymetrix.com/LinkServlet?probeset=203980_at) | 0.12 | 0.0055112 |
| Cyclin G2 | [CCNG2](http://www.ncbi.nlm.nih.gov/entrez/query.fcgi?cmd=search&db=gene&term=CCNG2) | [202769_at](https://www.affymetrix.com/LinkServlet?probeset=202769_at) | 0.22 | 0.0056679 |
| Nuclear factor of kappa light polypeptide gene enhancer in B-cells 2 (p49/p100) | [NFKB2](http://www.ncbi.nlm.nih.gov/entrez/query.fcgi?cmd=search&db=gene&term=NFKB2) | [209636_at](https://www.affymetrix.com/LinkServlet?probeset=209636_at) | 0.4 | 0.0058683 |
| Ellis van Creveld syndrome 2 | [EVC2](http://www.ncbi.nlm.nih.gov/entrez/query.fcgi?cmd=search&db=gene&term=EVC2) | [229974_at](https://www.affymetrix.com/LinkServlet?probeset=229974_at) | 0.39 | 0.0060125 |
| Calbindin 1, 28kDa | [CALB1](http://www.ncbi.nlm.nih.gov/entrez/query.fcgi?cmd=search&db=gene&term=CALB1) | [205625_s_at](https://www.affymetrix.com/LinkServlet?probeset=205625_s_at) | 0.29 | 0.0060957 |
| Spindle and kinetochore associated complex subunit 3 | [SKA3](http://www.ncbi.nlm.nih.gov/entrez/query.fcgi?cmd=search&db=gene&term=SKA3) | [227165_at](https://www.affymetrix.com/LinkServlet?probeset=227165_at) | 2.14 | 0.0062022 |
| HMG-box transcription factor 1 | [HBP1](http://www.ncbi.nlm.nih.gov/entrez/query.fcgi?cmd=search&db=gene&term=HBP1) | [209102_s_at](https://www.affymetrix.com/LinkServlet?probeset=209102_s_at) | 0.36 | 0.0062663 |
| Interleukin 13 receptor, alpha 2 | [IL13RA2](http://www.ncbi.nlm.nih.gov/entrez/query.fcgi?cmd=search&db=gene&term=IL13RA2) | [206172_at](https://www.affymetrix.com/LinkServlet?probeset=206172_at) | 0.24 | 0.006513 |
| Ral guanine nucleotide dissociation stimulator | [RALGDS](http://www.ncbi.nlm.nih.gov/entrez/query.fcgi?cmd=search&db=gene&term=RALGDS) | [209050_s_at](https://www.affymetrix.com/LinkServlet?probeset=209050_s_at) | 0.36 | 0.0065274 |
| Pyridine nucleotide-disulphide oxidoreductase domain 2 | [PYROXD2](http://www.ncbi.nlm.nih.gov/entrez/query.fcgi?cmd=search&db=gene&term=PYROXD2) | [228384_s_at](https://www.affymetrix.com/LinkServlet?probeset=228384_s_at) | 0.49 | 0.0065432 |
| Chromosome 17 open reading frame 49 | [C17orf49](http://www.ncbi.nlm.nih.gov/entrez/query.fcgi?cmd=search&db=gene&term=C17orf49) | [224574_at](https://www.affymetrix.com/LinkServlet?probeset=224574_at) | 0.46 | 0.006731 |
| Chromosome 8 open reading frame 48 | [C8orf48](http://www.ncbi.nlm.nih.gov/entrez/query.fcgi?cmd=search&db=gene&term=C8orf48) | [236634_at](https://www.affymetrix.com/LinkServlet?probeset=236634_at) | 2.11 | 0.0068043 |
| Ankyrin repeat domain 6 | [ANKRD6](http://www.ncbi.nlm.nih.gov/entrez/query.fcgi?cmd=search&db=gene&term=ANKRD6) | [204671_s_at](https://www.affymetrix.com/LinkServlet?probeset=204671_s_at) | 0.44 | 0.006806 |
| FUT8 antisense RNA 1 | [FUT8-AS1](http://www.ncbi.nlm.nih.gov/entrez/query.fcgi?cmd=search&db=gene&term=FUT8-AS1) | [236835_at](https://www.affymetrix.com/LinkServlet?probeset=236835_at) | 0.38 | 0.0069628 |
| Fibroblast growth factor 2 (basic) | [FGF2](http://www.ncbi.nlm.nih.gov/entrez/query.fcgi?cmd=search&db=gene&term=FGF2) | [204422_s_at](https://www.affymetrix.com/LinkServlet?probeset=204422_s_at) | 2.77 | 0.0070246 |
| Coiled-coil domain containing 138 | [CCDC138](http://www.ncbi.nlm.nih.gov/entrez/query.fcgi?cmd=search&db=gene&term=CCDC138) | [235644_at](https://www.affymetrix.com/LinkServlet?probeset=235644_at) | 2.22 | 0.0070442 |
| Protein phosphatase 4, regulatory subunit 1-like | [PPP4R1L](http://www.ncbi.nlm.nih.gov/entrez/query.fcgi?cmd=search&db=gene&term=PPP4R1L) | [223733_s_at](https://www.affymetrix.com/LinkServlet?probeset=223733_s_at) | 0.4 | 0.0071926 |
| MAX interactor 1 | [MXI1](http://www.ncbi.nlm.nih.gov/entrez/query.fcgi?cmd=search&db=gene&term=MXI1) | [202364_at](https://www.affymetrix.com/LinkServlet?probeset=202364_at) | 0.4 | 0.0072122 |
| Sequestosome 1 | [SQSTM1](http://www.ncbi.nlm.nih.gov/entrez/query.fcgi?cmd=search&db=gene&term=SQSTM1) | [213112_s_at](https://www.affymetrix.com/LinkServlet?probeset=213112_s_at) | 0.31 | 0.007387 |
| Hairy and enhancer of split 1, (Drosophila) | [HES1](http://www.ncbi.nlm.nih.gov/entrez/query.fcgi?cmd=search&db=gene&term=HES1) | [203395_s_at](https://www.affymetrix.com/LinkServlet?probeset=203395_s_at) | 0.33 | 0.0075505 |
| Fibroblast growth factor 1 (acidic) | [FGF1](http://www.ncbi.nlm.nih.gov/entrez/query.fcgi?cmd=search&db=gene&term=FGF1) | [1552721_a_at](https://www.affymetrix.com/LinkServlet?probeset=1552721_a_at) | 2.65 | 0.0075994 |
| Exonuclease 3'-5' domain containing 3 | [EXD3](http://www.ncbi.nlm.nih.gov/entrez/query.fcgi?cmd=search&db=gene&term=EXD3) | [241363_at](https://www.affymetrix.com/LinkServlet?probeset=241363_at) | 0.3 | 0.0077245 |
| Hydroxysteroid (17-beta) dehydrogenase 14 | [HSD17B14](http://www.ncbi.nlm.nih.gov/entrez/query.fcgi?cmd=search&db=gene&term=HSD17B14) | [224494_x_at](https://www.affymetrix.com/LinkServlet?probeset=224494_x_at) | 0.36 | 0.0080065 |
| Myotubularin related protein 11 | [MTMR11](http://www.ncbi.nlm.nih.gov/entrez/query.fcgi?cmd=search&db=gene&term=MTMR11) | [205076_s_at](https://www.affymetrix.com/LinkServlet?probeset=205076_s_at) | 0.45 | 0.0081785 |
| Msh homeobox 1 | [MSX1](http://www.ncbi.nlm.nih.gov/entrez/query.fcgi?cmd=search&db=gene&term=MSX1) | [205932_s_at](https://www.affymetrix.com/LinkServlet?probeset=205932_s_at) | 0.36 | 0.0082241 |
| Chemokine (C-X-C motif) ligand 2 | [CXCL2](http://www.ncbi.nlm.nih.gov/entrez/query.fcgi?cmd=search&db=gene&term=CXCL2) | [209774_x_at](https://www.affymetrix.com/LinkServlet?probeset=209774_x_at) | 0.15 | 0.0082372 |
| Nexilin (F actin binding protein) | [NEXN](http://www.ncbi.nlm.nih.gov/entrez/query.fcgi?cmd=search&db=gene&term=NEXN) | [226103_at](https://www.affymetrix.com/LinkServlet?probeset=226103_at) | 2.16 | 0.0083034 |
| Gamma-aminobutyric acid (GABA) B receptor, 1 | [GABBR1](http://www.ncbi.nlm.nih.gov/entrez/query.fcgi?cmd=search&db=gene&term=GABBR1) | [203146_s_at](https://www.affymetrix.com/LinkServlet?probeset=203146_s_at) | 0.48 | 0.0083621 |
| NEDD4 binding protein 2-like 1 | [N4BP2L1](http://www.ncbi.nlm.nih.gov/entrez/query.fcgi?cmd=search&db=gene&term=N4BP2L1) | [213375_s_at](https://www.affymetrix.com/LinkServlet?probeset=213375_s_at) | 0.35 | 0.0087295 |
| Abhydrolase domain containing 13 | [ABHD13](http://www.ncbi.nlm.nih.gov/entrez/query.fcgi?cmd=search&db=gene&term=ABHD13) | [234993_at](https://www.affymetrix.com/LinkServlet?probeset=234993_at) | 2.53 | 0.0087343 |
| ABI family, member 3 (NESH) binding protein | [ABI3BP](http://www.ncbi.nlm.nih.gov/entrez/query.fcgi?cmd=search&db=gene&term=ABI3BP) | [223395_at](https://www.affymetrix.com/LinkServlet?probeset=223395_at) | 0.31 | 0.0087609 |
| Histone deacetylase 9 | [HDAC9](http://www.ncbi.nlm.nih.gov/entrez/query.fcgi?cmd=search&db=gene&term=HDAC9) | [205659_at](https://www.affymetrix.com/LinkServlet?probeset=205659_at) | 0.37 | 0.0088992 |
| Collagen triple helix repeat containing 1 | [CTHRC1](http://www.ncbi.nlm.nih.gov/entrez/query.fcgi?cmd=search&db=gene&term=CTHRC1) | [225681_at](https://www.affymetrix.com/LinkServlet?probeset=225681_at) | 0.21 | 0.0090321 |
| 1-Acylglycerol-3-phosphate O-acyltransferase 4 (lysophosphatidic acid acyltransferase, delta) | [AGPAT4](http://www.ncbi.nlm.nih.gov/entrez/query.fcgi?cmd=search&db=gene&term=AGPAT4) | [219693_at](https://www.affymetrix.com/LinkServlet?probeset=219693_at) | 0.45 | 0.0095554 |
| Glutamine-fructose-6-phosphate transaminase 2 | [GFPT2](http://www.ncbi.nlm.nih.gov/entrez/query.fcgi?cmd=search&db=gene&term=GFPT2) | [205100_at](https://www.affymetrix.com/LinkServlet?probeset=205100_at) | 0.39 | 0.009563 |
| Related RAS viral (r-ras) oncogene homolog | [RRAS](http://www.ncbi.nlm.nih.gov/entrez/query.fcgi?cmd=search&db=gene&term=RRAS) | [212647_at](https://www.affymetrix.com/LinkServlet?probeset=212647_at) | 0.41 | 0.0098394 |
| Kruppel-like factor 9 | [KLF9](http://www.ncbi.nlm.nih.gov/entrez/query.fcgi?cmd=search&db=gene&term=KLF9) | [203542_s_at](https://www.affymetrix.com/LinkServlet?probeset=203542_s_at) | 0.36 | 0.0099051 |
